# Surviving the host: Microbial metabolic genes required for growth of *Pseudomonas aeruginosa* in physiologically-relevant conditions

**DOI:** 10.3389/fmicb.2022.1055512

**Published:** 2022-11-25

**Authors:** Corrie R. Belanger, Melanie Dostert, Travis M. Blimkie, Amy Huei-Yi Lee, Bhavjinder Kaur Dhillon, Bing Catherine Wu, Noushin Akhoundsadegh, Negin Rahanjam, Javier Castillo-Arnemann, Reza Falsafi, Daniel Pletzer, Cara H. Haney, Robert E. W. Hancock

**Affiliations:** ^1^Centre for Microbial Diseases and Immunity Research, Department of Microbiology and Immunology, University of British Columbia, Vancouver, BC, Canada; ^2^Department of Molecular Biology and Biochemistry, Simon Fraser University, Burnaby, BC, Canada; ^3^Department of Microbiology and Immunology, University of Otago, Dunedin, New Zealand; ^4^Department of Microbiology and Immunology, University of British Columbia, Vancouver, BC, Canada

**Keywords:** *Pseudomonas aeruginosa*, host-like media, *in vivo* survival, murine abscess model, human skin organoid model

## Abstract

*Pseudomonas aeruginosa,* like other pathogens, adapts to the limiting nutritional environment of the host by altering patterns of gene expression and utilizing alternative pathways required for survival. Understanding the genes essential for survival in the host gives insight into pathways that this organism requires during infection and has the potential to identify better ways to treat infections. Here, we used a saturated transposon insertion mutant pool of *P. aeruginosa* strain PAO1 and transposon insertion sequencing (Tn-Seq), to identify genes conditionally important for survival under conditions mimicking the environment of a nosocomial infection. Conditions tested included tissue culture medium with and without human serum, a murine abscess model, and a human skin organoid model. Genes known to be upregulated during infections, as well as those involved in nucleotide metabolism, and cobalamin (vitamin B_12_) biosynthesis, etc., were required for survival *in vivo*- and in host mimicking conditions, but not in nutrient rich lab medium, Mueller Hinton broth (MHB). Correspondingly, mutants in genes encoding proteins of nucleotide and cobalamin metabolism pathways were shown to have growth defects under physiologically-relevant media conditions, *in vivo, and in vivo-*like models, and were downregulated in expression under these conditions, when compared to MHB. This study provides evidence for the relevance of studying *P. aeruginosa* fitness in physiologically-relevant host mimicking conditions and identified metabolic pathways that represent potential novel targets for alternative therapies.

## Introduction

*Pseudomonas aeruginosa* is a highly drug resistant, hospital-acquired, opportunistic pathogen that thrives in the restricted nutrient environments within the host. This organism can rapidly alter its gene expression patterns, surviving with limited resources despite the onslaught of defence mechanisms by the host (Fung et al., 2010; Kruczek et al., 2016; Quinn et al., 2018). For example, *P. aeruginosa* displays altered transcriptional regulation in physiologically-relevant media conditions mimicking growth in wound exudate or blood (Belanger et al., 2020). Wounds and blood infections in immunocompromised patients represent a substantial proportion of hospital-acquired *P. aeruginosa* infections and can critically lead to sepsis (Hattemer et al., 2013). In this regard, determining differences in bacterial physiology and susceptibility under conditions that are relevant to human infections is crucial to advance our understanding of how to treat drug resistant infections by ESKAPE (*Enterococcus faecium*, *Staphylococcus aureus*, *Klebsiella pneumoniae*, *Acinetobacter baumannii*, *P. aeruginosa*, *Enterobacter* sp.) pathogens.

RNA-Seq has proven to be a useful tool to explore the bacterial physiology and altered gene expression that occurs in different growth environments such as blood (Malachowa et al., 2011; Kruczek et al., 2016), serum (Hammond et al., 2010; Kruczek et al., 2014; Quinn et al., 2018), lung sputum (Fung et al., 2010), and wound models (Turner et al., 2014). Studies have used minimal medium supplemented with bovine serum or albumin to show that serum can inhibit short term biofilm formation on indwelling devices (Hammond et al., 2010), and that iron-regulated bacterial virulence genes are also upregulated in response to serum albumin (Kruczek et al., 2012). Turner et al. (2014) analyzed the transcriptome of *P. aeruginosa* in two murine models, an acute dermal burn wound model and a chronic excision wound infection model. They found that many virulence genes such as LPS O-antigen biosynthesis genes were downregulated *in vivo* when compared to defined minimal medium MOPS, while pyoverdine synthesis and T3SS genes were upregulated *in vivo*. Furthermore, rowth of *P. aeruginosa* in tissue culture medium with human serum can alter the expression of ~39% of the genome when compared to growth in the nutrient-rich laboratory medium, Mueller Hinton Broth (MHB) (Belanger et al., 2020). This altered gene expression in physiologically-relevant media appeared to increase membrane permeability and susceptibility to the antibiotic azithromycin. More profoundly, we previously demonstrated that the global transcriptome of *P. aeruginosa* grown in tissue culture medium with human serum displayed significant overlap with the transcriptomes of *P. aeruginosa* from wound and lung infections (Belanger et al., 2020; Cornforth et al., 2020). This finding demonstrated that these media represent an easy screening method for determining how gene expression changes with altered growth or susceptibility in physiologically relevant conditions. Although it can infer a great deal about an organism’s physiology and how the organism changes its gene expression in response to an environmental cue, RNA-Seq cannot be used to determine which genes are required or important for survival of a bacterium under particular conditions. Instead, a method called transposon insertion sequencing (Tn-Seq) can be used to identify genes that are important for conditional fitness of an organism, including genes that, when mutated, cause lethal growth defects under physiologically-relevant conditions (Skurnik et al., 2013; Chao et al., 2016; Poulsen et al., 2019).

Tn-Seq utilizes a promiscuous transposon (Tn) to create saturated pools of Tn-insertion mutants, that can be grown in selective or challenged conditions to eliminate mutants with decreased fitness in the environment of choice (Chao et al., 2016; Cain et al., 2020). Sequencing of the regions adjacent to the Tn for each mutant that survives in the pool after selection, determines which mutants were eliminated, and indicates that the eliminated genes were required for growth in the condition of interest. For example, genes that are considered essential in an organism grown in MHB are not necessarily required under conditions such as wound exudate or infected blood, and vice versa. Such conditional essentiality means that genes required for growth in, e.g., the host but not in rich lab media, might be strong candidates as targets for therapeutics that have not previously been discovered. However, such genes cannot be discovered by studying essentiality under standardized nutrient rich conditions.

There are several *in vivo* models designed to mimic human infections such as wounds (Bobrov et al., 2022). Burn wound, and chronic wound/skin abscess animal models have been adapted for use with all ESKAPE pathogens (Turner et al., 2014; Pletzer et al., 2017a) and offer relatively simple primary screening methods for the establishment and/or treatment of bacterial infections. In wound/abscess models, bacteria are injected into the subdermal tissue below the skin or to skin that has been previously burned. By treating the abscess or injecting various strains of an organism, the resulting effects on abscess size and colony forming units (CFU) of bacteria in the abscess can help screen for effective antimicrobial agents to treat chronic infections, or for bacterial survival requirements in chronic infections (Turner et al., 2014; Mansour et al., 2016; Pletzer et al., 2017a,b). Additionally, *in vitro* organoid or air-liquid interface models such as a recently-described human skin organoid model are addressing some of the cost-associated and ethical concerns of animal models by using human cell lines or cells directly obtained from patients (de Breij et al., 2018; Wu et al., 2021). In this humanized system, keratinocytes are differentiated *in vitro* into dermal tissues that are infected with bacteria and can be treated topically with antimicrobial agents. Alternatively, *in vitro* physiologically relevant media conditions represent a method to screen for survival and susceptibility of pathogens while requiring much less cost and time to perform than *in vivo* and *in vivo*-like models. Media such as Roswell Park Memorial Institute 1,640 medium (RPMI) and RPMI with human serum represent a means to study *P. aeruginosa* in an environment meant to mimic human infection and the applicability of these conditions to host infection has been demonstrated in RNA-Seq studies (Belanger et al., 2020). Tn-Seq studies in these media conditions could further validate their use as physiologically-relevant growth environment, while providing an affordable and easy screening method to discover novel therapeutic targets specific to the host environment.

Here we designed and utilized Tn-Seq to identify genes required *in vitro* and determined how similar fitness determinants in host mimicking conditions were to an *in vivo* environment. *In vitro* physiologically relevant media RPMI and RPMI with human serum were chosen to screen for potential pathways that are essential for survival in nosocomial wound or blood infection. Genes unique to these media compared to nutrient rich lab media, were subsequently compared to conditionally important genes in the murine abscess model and the human skin organoid model. We identified functional pathways that were uniquely important for survival under host mimicking *in vitro* conditions and *in vivo*, to inform upon how *P. aeruginosa* survives during wound infections in ways that are different from survival under laboratory conditions. We report on the characterization of the Tn-mutant pool in *P. aeruginosa* PAO1 and the identification of genes required for survival under these physiologically-relevant conditions. This study has allowed us to identify genes in key pathways that impact on survival under a range of conditions mimicking the host infectious environment, and has implications for *in vitro* screening and identification of novel targets for therapeutics.

## Materials and methods

### Strains and bacterial growth conditions

*P. aeruginosa* PAO1 strain H103 (Zhang et al., 2000) was used as a WT control and for Tn-Seq studies. The Tn-insertion mutants used for susceptibility and growth defect validations were taken from an ordered Tn-insertion library in *P. aeruginosa* PAO1 (harboring Tn5 IS50L derivative Tn-insertions IS*lacZ*/hah and IS*phoA*/hah; Jacobs et al., 2003). *E. coli* strain SM10 λpir was used as a donor strain in Tn-Seq pool generation.

Media included lysogeny broth (LB), experimental control condition MHB, RPMI-1640 supplemented with 5% MHB (referred to as RPMI), RPMI-1640 supplemented with 5% MHB and 20% human serum pooled from anonymous donors under ethics approval [certificate number H04-70232]. RPMI and RPMI/serum were designed to be physiologically-relevant to wound exudate or blood and their preparation was described in detail previously (Belanger and Hancock, 2021).

### Plasmids and DNA constructs

The pBT20 plasmid was used for generation of the Tn-Seq pool and was obtained from the Filloux lab, Imperial College, London (Kulasekara et al., 2005). This is a mariner Tn vector with the Himar1 Tn that randomly inserts into Thymine-Adenine (TA) sites within the genome. The plasmid has the following characteristics: Δ*bla* Tel r; The Tel r cassette, consisting of the *kilA* and *telAB* genes, is flanked by two identical FRT sequences. Primers used in this study are summarized in Supplementary Table S1.

### Constructing the Tn-Seq pool in PAO1

A Tn-Seq pool was constructed in *P. aeruginosa* strain PAO1 using the vector pBT20. First, *E. coli* SM10λpir + pBT20 was grown overnight at 37°C on LB agar containing 100 μg/ml ampicillin (Sigma-Aldrich). *P. aeruginosa* PAO1 was grown at 42°C overnight on LB agar with no antibiotic. The bacteria were scraped off the plates and mixed in equal volumes of each at a ratio of OD_600_ 40:20 donor (*E. coli*): recipient (*P. aeruginosa*). Conjugation mixtures were spotted on to LB agar plates and incubated for 2 h at 37°C. All conjugation spots were then scraped into LB and plated on LB agar with 25 μg/ml irgasan (Sigma-Aldrich) and 25 μg/ml gentamicin (Sigma-Aldrich). The cells were grown overnight then counted and collected in LB, and stored in 20% glycerol at −80°C. The procedure was repeated until at least 200,000 mutants were collected. The collected mutants were thawed at 37°C, normalized to equalize the number of colonies per mL per conjugation, and then pooled and recovered at 37°C. They were recovered by adding 0.5 ml of pooled cells into 50 ml LB with 15 μg/ml gentamicin. 20% glycerol was added to the final pool and it was aliquoted into 1 ml volumes and flash frozen with liquid nitrogen before storing at −80°C.

### Preparation of the Tn-Seq libraries for sequencing

To determine which mutants existed in the Tn-Seq pool at baseline and after any selective pressure, total genomic DNA was extracted from the samples and the regions adjacent to the Tn-insertions were amplified and sequenced. Primers used for Tn-Seq are summarized in Supplementary Table S1. First, total DNA was extracted using the DNeasy Blood and Tissue Kit (QIAGEN). The concentration and purity of the DNA was measured using a NanoDrop 2000 (Thermo Fisher). Genomic DNA was used in four replicate PCR reactions to amplify the Tn-genome junctions *via* two nested PCR steps which are described in detail in Supplementary Materials. Products of replicate reactions of the second PCR step were pooled together and two-sided size selection was performed with Agencourt AMPure XP magnetic beads. DNA from each pool was eluted into 40 μl total in nuclease free Tris-EDTA buffer. Four aliquots of 5 μl were used for indexing and sequencing. The indexing PCR was done according to the Illumina protocol for amplicon sequencing using Nextera Index Kit v2 SetA (Illumina). Replicates were pooled and a final PCR cleanup was performed with Agencourt AMPure XP magnetic beads with elution into 40 μl nuclease free TE buffer. Libraries were quantified using Quant-iT dsDNA Assay Kit (Thermo Fisher) analyzed on the Bioanalyzer 2,100 (Agilent Genomics). Samples were diluted to 4 nM and pooled with other samples for an HiSeq sequencing lane, at 3–6% of Tn-Seq library total per lane. Samples were run on a HiSeq 2,500 single end 100 bp run (Illumina).

### Bioinformatic analysis of Tn-Seq results

The quality of raw sequence reads was assessed using FastQC (v0.11.6; Andrews, 2010) and MultiQC (v1.6; Ewels et al., 2016). Conditionally essential genes were determined using two, complementary bioinformatics tools: TraDIS v1.4.5 (referred to as Tradis; Barquist et al., 2016) and TRANSIT v2.0.0 (Transit; DeJesus et al., 2015). The sequence reads were aligned against *P. aeruginosa* PAO1 reference genome from the *Pseudomonas* Genome Database v17.1 (accession GCF_000006765.1; PseudoCap version 138). Tradis was run using default parameters by aligning reads against the reference genome using SMALT and determining insertion counts per gene. Transit used TPP pre-processing tool to map raw reads against the reference with BWA v0.7.17 (Li and Durbin, 2009) with default parameters used to tabulate the counts of reads per TA sites. The counts were passed through the Gumbel method of Transit for calculating the probability of essentiality of each gene using a Bayesian model. Genes predicted to be essential by Gumbel or Tradis in at least 4 out of 5 replicates (in experimental replicates) or 2 out of 3 replicates (for the T0 pool) were compiled into a final list of essential genes.

Statistical significance of overlap between the Tn-Seq pool selected in different physiologically-relevant media was performed using hypergeometric distribution calculations and Fisher’s exact test with the GeneOverlap v1.32.0 package from BioConductor release 3.15 (Shen and Sinai, 2022) in R v4.1.2 (R Core Team, 2021). Overlaps in essential genes found between two physiologically-relevant media conditions was considered significant with an odds ratio greater than 1 if *p* < 0.05.

Functional class enrichment was performed using functional class annotations from PseudoCap (Winsor et al., 2016) and hypergeometric distribution calculations were done using phyper from the stats package v4.3.0 in R (Johnson et al., 1992; R Core Team, 2021) and correcting for multiple testing using false discovery rate (FDR) corrections. Functional classes considered significantly enriched within a comparison were those with adjusted *p* value <0.05.

### Experiments in physiologically-relevant *in vitro* conditions

Tn-insertion pools were inoculated at 1 × 10^7^ CFU/ml in 25 ml cultures in MHB, RPMI and RPMI/serum in five separate biological replicates performed over multiple days. The cultures were grown to 5 × 10^8^ CFU/ml at 37°C and bacteria were harvested from 1 ml each. Total genomic DNA was extracted, libraries were prepared and sequenced, and essentiality analysis was performed using Tradis and Transit. Conditionally essential genes were compared between media (physiologically-relevant conditions vs. MHB), and essential genes in MHB were removed from the physiologically-relevant media gene lists before comparing to other host mimicking conditions.

Validation experiments were performed with individual Tn-insertion mutants grown in physiologically-relevant *in vitro* media in 96-well plates. The wells containing RPMI/serum or MHB were inoculated with minimum 3 replicates of each mutant in each medium at 1 × 10^7^ CFU/ml and grown for 16 h in a plate reader (BioTek) with constant shaking and heating to 37°C. Select mutants with interesting growth defects were then grown in 25 ml flask cultures in RPMI/serum. Cultures were grown at 37C for 25 h and samples were taken and plated for CFU every 3 h for the first 9 h and then at the final time point. Growth cultures were performed with PAO1 WT grown in parallel to mutant strains.

### Murine abscess model experiments

Animal experiments were performed following Canadian Council on Animal Care (CCAC) guidelines and were approved by The University of British Columbia Animal Care Committee [certificate number A14-0363]. The mice used in this study were seven-week-old female outbred CD-1, purchased from Charles River Laboratories, Inc., (Wilmington, MA). The average weight was about 25 ± 3 g at the time of the experiments. One to 3% isoflurane was used to anesthetize the mice. Mice were euthanized with carbon dioxide.

For murine abscess Tn-Seq an aliquot of the PAO1 Tn-Seq pool was grown to mid log phase, washed, and resuspended in PBS to and OD_600_ of 0.5. The pool was administered to mice subcutaneously with a total bacterial inoculum of 2 × 10^7^ CFU. Abscesses were allowed to form for 18 h, at which time the mice were sacrificed, and the abscesses removed. The experiment was repeated with five replicates.

Enzymatic digestion of the abscess tissue and separation of bacterial cells followed a previously published protocol with modifications (Garcia-Garcerà et al., 2013). The abscess tissue was homogenized in 1 mL sterile PBS with sterile glass beads and 10 μl were removed for CFU counting. The tissue was then enzymatically digested at 37°C for 45 min, inactivated and filtered. The final, filtered, washed cells were pelleted and used for DNA extraction using the same method as for Tn-Seq in host mimicking *in vitro* conditions. Library prep, sequencing and essentiality analysis was performed as described above.

Survival of ordered Tn-insertion mutants for genes identified through Tn-Seq in the murine abscess model was performed in a similar fashion to Tn-Seq experiments.

### Human skin air-liquid interface model experiments

Tn-Seq was also performed in a human skin *in vivo*-like model (Wu et al., 2021). The human skin organoid (skin) model used N/TERT keratinocyte cells cultured on filter inserts in 12-well plates containing growth factors and tissue culture medium. Over approximately 3 weeks, the cells grew, differentiated, and stratified into a dermal-like tissue on which bacteria could be inoculated.

The Tn pool was grown in LB to mid log phase, washed with PBS, and inoculated onto the skin at 1 × 10^6^ CFU in five μL of PBS and allowed to develop for 48 h. The skin was then washed with PBS, and the entire filter with skin and bacteria attached was removed from the tissue culture wells, homogenized, and plated to measure survival. The skin was then digested following the same protocol as for abscess tissue. Whole genomic DNA extraction was performed and the Tn-genome junctions were amplified and sequenced as above. The experiment was repeated with four replicates from different skin batches.

Competition between PAO1 ordered Tn-insertion mutants for genes identified through Tn-Seq and wild type (WT) PAO1 was measured after 48 h of bacterial growth on the skin. A control Tn-insertion mutant was included where the insertion is at the end of the general essential gene *dnaG* and thus does not demonstrate an insertional effect and would not be expected to have a growth advantage or disadvantage when compared to WT. The skin was inoculated with 1 × 10^6^ CFU in 5 μl of PBS containing 50% WT and 50% mutant population. The infection was left for 48 h, then the skins were washed with sterile PBS, homogenized using glass beads, and plated on LB agar (for growth of all bacteria) and tetracycline (50 μg/ml: for growth of Tn-insertion mutants). The ratios of mutant to WT were measured for all mutants tested (Macho et al., 2007). Competitive fitness indices (CI) were assessed using the following formula CI = (M_O_/WT_O_)/(M_I_/WT_I_), where, M was the CFU/mL of the mutant, WT was the CFU/mL of the wild type, subscript O is the output counts after a biofilm was established on the skin, and subscript I was the input counts in the inoculum.

### Network analysis of Tn-Seq and RNA-Seq data

The web-based application PaintDB (Castillo-Arnemann et al., 2021) was utilized to create networks in order to visualize RNA-Seq and Tn-Seq data together. To explore genes essential under physiologically-relevant conditions and the transcriptional patterns of genes in the same pathways, RNA-Seq data for *P. aeruginosa* grown in RPMI/serum (Belanger et al., 2020) was combined with the gene list of genes conditionally essential in RPMI and/or RPMI/serum. Subnetworks of significantly enriched pathways were exported and edited for visualization using Cytoscape (v 3.8.2; Shannon, 2003). Using the ontologies tool in PaIntDB, subnetworks were constructed for pathways that were significantly enriched in these datasets using Fischer’s exact test with Bonjamini/Hochberg correction and a 0.05 significance threshold.

## Results

### Characterization of the PAO1 Tn-Seq pool determined generally essential genes are comparable to previous studies

A Tn-Seq pool was generated in *P. aeruginosa* strain PAO1 grown in LB, using the mariner Tn Himar1 that randomly inserts into TA sites within the genome (Lampe et al., 1998). Over 200,000 mutants were collected, to generate a Tn pool that was more than two-fold larger than the possible insertion sites in the genome. To identify essential genes in LB, before expansion experiments were performed, three individual aliquots (biological replicates) of the Tn-insertion pool (denoted as T0) were sequenced. On average 4.4 million reads mapped to the genome, with between 90,000 and 100,000 unique insertions sites (Supplementary Dataset 1). Nineteen genes were excluded from the analysis as they contained no TA sites (Supplementary Dataset 1). To determine essential genes, a pipeline was constructed to utilize the analysis applications Tradis (Barquist et al., 2016), which determined essentiality by assessing insertions per gene, and Transit (DeJesus et al., 2015), which calculated the probability of essentiality of each gene using reads per TA site and a Bayesian model. By integrating two distinct analysis tools, the pipeline took into account variation in results that might occur due to differing algorithms in an attempt to reduce the risk of reproducibility issues. Genes were termed essential if they were identified as essential by either tool in at least 2 out of 3 replicate aliquots. A total of 654 genes were predicted as essential in the T0 pool in this study.

To analyze the robustness of the Tn-Seq pool, the essential genes identified in our experiment were compared to essential genes identified in previous Tn-Seq and ordered Tn mutant studies in *P. aeruginosa*, to assess similarities between the libraries (Table 1; Supplementary Dataset 1). Previously, investigations of *P. aeruginosa* essentiality have predicted between 336 and 1,394 generally essential genes when this organism is grown under rich growth media conditions (Table 1; Liberati et al., 2006; Skurnik et al., 2013; Lee et al., 2015; Turner et al., 2015; Poulsen et al., 2019). Overall, there were 147 genes predicted as essential in rich medium in our T0 pool, as well as in all six published essentiality studies (Supplementary Dataset 1). In addition to those predicted in all studies, another 135 were essential in the T0 pool and in five other essentiality studies, for a total of 282 common, frequently identified essential genes. Using hypergeometric distribution statistics, we determined that our T0 essential genes had significant overlap with all previous studies examined, with the highest correlation with Lee et al. (2015), Poulsen et al. (2019), and Turner et al. (2015) (Supplementary Dataset 1). Additionally, we found 10/26 Pseudocap functional classes (Winsor et al., 2016) showed common significant enrichment of essential genes in five, six, or seven of the studies examined here (Supplementary Table S2). These results indicate that the bacterial growth condition or method used to determine essentiality might strongly impact on the number and categories of genes identified as essential. Despite this, we determined significant overlapping functional classes to previous studies, and found our T0 pool to be a good representative of generally essential genes of *P. aeruginosa* in nutrient rich LB.

**Table 1:** Numbers of essential genes in nutrient rich medium as predicted in this and other studies on *P. aeruginosa* strains PAO1 and PA14 using various analysis methods.

Strain	Media	Method	Predicted essential genes	Study
PAO1	LB	Transit (calculating the probability of essentiality of each gene using reads/Thymine-Adenine (TA) site and a Bayesian model; Output is Essential or Nonessential) using Himar1 Tn.	229	This study
PAO1	LB	Tradis (reads/gene; genes with 0 reads in n-1/n replicates are considered essential) using Himar1 Tn.	630	This study
PAO1	LB	Reads/Kb and hits/Kb (essential in n-1/n replicates) using ISlacZhah-Tc Tn.	453	Lee et al. (2015)
PAO1	LB	Monte Carlo simulations & DESeq (observed Tn insertions vs. expected Tn insertions) using ISlacZhah-tc Tn.	336	Turner et al. (2015)
PA14	BHI	Monte Carlo simulations & DESeq (observed Tn insertions vs. expected Tn insertions) using ISlacZhah-tc Tn.	435	Turner et al. (2014)
PA14	LB	Genes with fewer than 10 reads in Tn pool using Himar1 Tn.	636	Skurnik et al. (2013)
PA14	LB	FiTnEss (reads/TA site calculated using Transit; compared distribution to theoretical distribution) calculated using Tn-Seq library prepared with derivative of *MAR2xT7*	599	Poulsen et al. (2019)
PA14	LB	Genes not disrupted in ordered *MAR2xT7* mutant libraries	1,394	Liberati et al. (2006)

### Tn-Seq of *Pseudomonas aeruginosa* grown under *in vitro* and *in vivo*/*in vivo*-like conditions identified significant overlap in conditionally required genes between host mimicking conditions

Following the characterization of the PAO1 Tn-insertion pool, this library was used to determine which genes were conditionally important for survival in physiologically-relevant *in vitro* media (Belanger et al., 2020), in an *in vivo* murine abscess model (Pletzer et al., 2017b), and in an *in vivo*-like human skin organoid model (Wu et al., 2021). From this, we aimed to understand whether genes required for survival of *P. aeruginosa* in host mimicking media were also required in established abscess and skin models, and to discover unexplored pathways that might be important for survival in wound models. By focusing on genes determined to be conditionally required in each medium (mutants completely absent after expansion) and excluding genes contributing to fitness but not absolutely required (mutants with decreasing numbers), we identified genes that were likely impacting to the greatest extent on survival and fitness in the host environment.

#### *In vitro* in physiologically relevant media conditions

We grew the Tn-Seq pool in RPMI and RPMI/serum as *in vitro* mimics of physiologically-relevant conditions, as well as in MHB as a nutrient rich control. The media was inoculated with 1 × 10^7^ CFU/ml and grown until mid-log phase (5 × 10^8^ CFU/ml), with the final bacterial concentration representing 1,000-fold the number of mutants present in the T0 pool. Five replicates of the pool were grown in the physiologically relevant media and MHB and genes were deemed essential if they were predicted by either Tradis or Transit in at least four replicates. After growth in physiologically-relevant *in vitro* media there were 280 conditionally essential genes that were not identified as essential for growth in MHB (Supplementary Figure S1; Supplementary Dataset 2A). Of these 280 genes, 70 were predicted to be conditionally essential for survival in both RPMI/serum and RPMI (Supplementary Table S3), 27 were specifically important for survival in RPMI/serum only, and 183 genes were important for survival in RPMI only. Importantly, the genes that were essential in either physiologically relevant medium were largely belonging to the same PseudoCap (Winsor et al., 2016) functional classes (Supplementary Figure 1B). These data suggested that serum components may provide important factors affecting survival in host conditions, but that RPMI alone is a more nutrient exacting condition, leading to identification of a greater number of conditionally important genes. The combined 280 gene set for essentiality in host mimicking *in vitro* media, was used in further comparative analyses to other host-like conditions.

#### Murine abscess model

**Figure 1:**
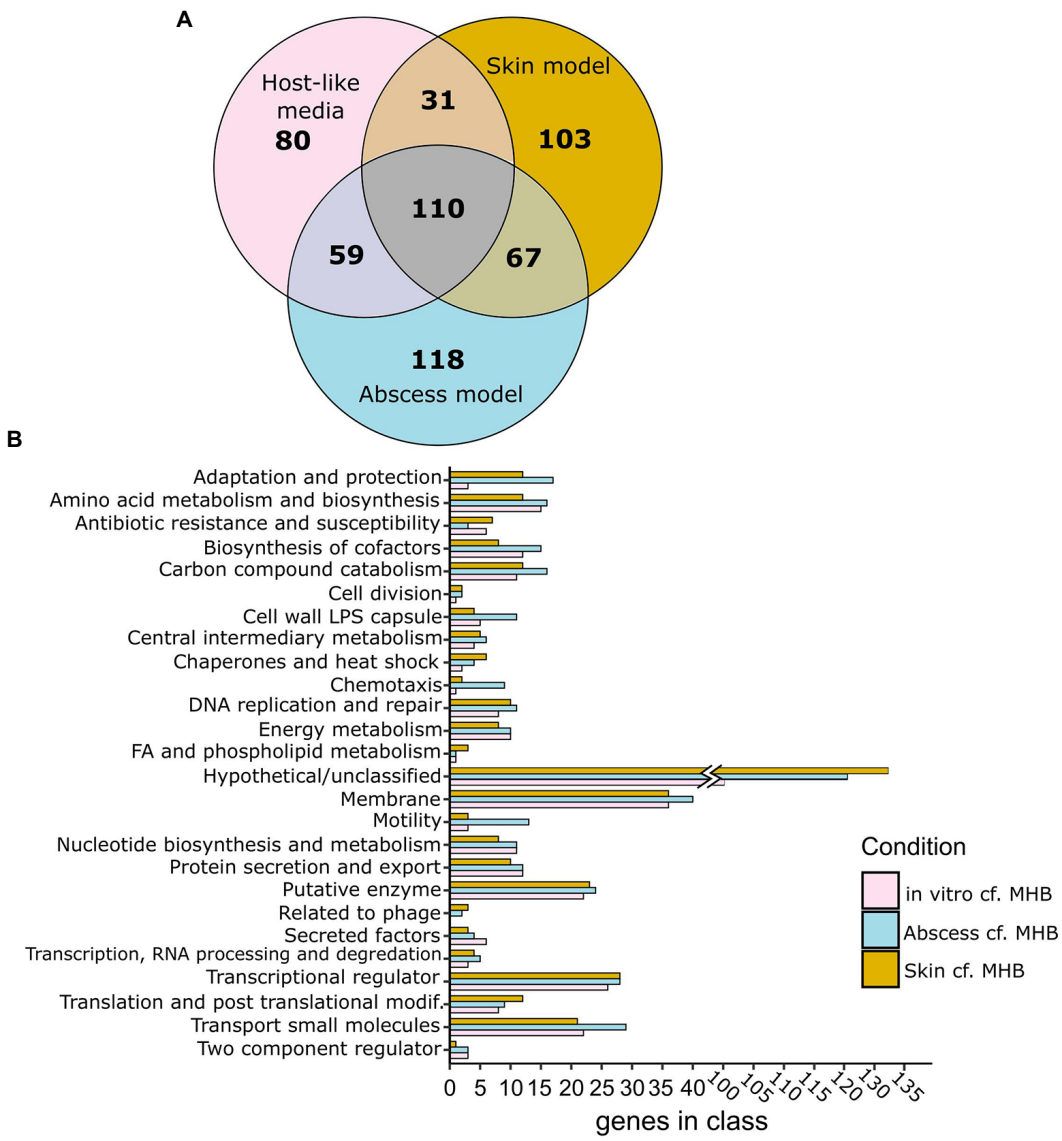
Genes predicted as conditionally essential for growth in murine abscess and human skin models and in physiologically-relevant media (RPMI and or RPMI/Serum) *cf.* MHB as determined using Tn-Seq. **(A)** Venn diagrams showing essential genes that are unique or shared between particular host-like conditions (RPMI or RPMI/Serum: pink; human skin organoid model: amber; murine abscess model: blue), as determined by either Tradis or Transit *cf.* MHB. **(B)** Functional classes of essential genes of *Pseudomonas aeruginosa* grown *in vitro* (in RPMI and/or RPMI/Serum), murine abscesses, or human skin *cf.* MHB.

Next, genes conditionally important for survival *in vivo* were determined using the murine abscess model of *P. aeruginosa* infection (Pletzer et al., 2017b). The Tn-Seq pool was injected at an inoculum of 2 × 10^7^ CFU into subdermal tissue of a mouse and an abscess was established for 18 h, resulting in an average of 2 × 10^9^ CFU/abscess. To determine if genes important for survival in the murine abscess model were also important in the physiologically-relevant *in vitro* media conditions, we compared essential genes between each condition, excluding genes identified as essential in MHB. Among 354 genes conditionally essential for *P. aeruginosa* survival in the murine abscess model and 280 in physiologically-relevant *in vitro* media conditions, we identified 169 genes that were essential in both (Figure 1A; Supplementary Dataset 2B). This overlap was determined to be highly significant (*p* = 4.6 × 10^−146^, Fisher’s exact test) with an odds ratio of 43.0.

#### Human skin organoid model

Tn-Seq was also performed to identify predicted essential genes in *P. aeruginosa* PAO1 grown on a human skin organoid model (Wu et al., 2021). The PAO1 Tn-Seq pool was inoculated at 1 × 10^6^ CFU onto the surface of a differentiated dermal tissue layer on a filter insert in a tissue culture plate. Low inoculation concentrations were used in order to prevent *P. aeruginosa* from penetrating the tissue layer and growing in the medium below. Only experiments where *P. aeruginosa* cultures remained on the surface of the skin were included in the analysis. Bacteria growing on the skin after 48 h (on average 4 × 10^7^ CFU) were harvested, and their DNA was extracted, and Tn-genome junctions sequenced. We compared essential genes between the skin model and other physiologically-relevant models with genes essential in MHB excluded to identify genes specifically important in the host. There were 311 genes found to be conditionally important in the human skin model, *cf.* MHB, 141 of which were also identified in physiologically-relevant *in vitro* media conditions (Odds Ratio of 31.3, *p* = 1.5 × 10^−113^, Fisher’s exact) and 177 of which were also essential in the abscess model (Odds Ratio of 38.8, p = 1.5 × 10^−147^, Fisher’s exact; Figure 1A; Supplementary Dataset 2C).

These data support that there is significant overlap between genes required for survival in *in vitro* host mimicking conditions and *in vivo*. In the next sections, we contrasted and compared functional classes of genes identified in common between the host mimicking conditions to explore important functions for survival in the host environment.

### Genes unique to physiologically-relevant *in vitro* or *in vivo* media might represent limitations of *in vitro* host mimicking conditions

Comparing predicted essential genes lists between conditions, we found there were 67 genes identified as conditionally important for survival in both abscess and the skin model that were not identified in RPMI or RPMI/serum. Interestingly, this included a larger number of genes involved in adaptation and protection, chaperones and heat-shock proteins, transcription, translation and DNA repair (Figure 1B; Supplementary Table S4). Among the genes involved in adaptation and protection that were found only in the murine abscess and skin models were the type VI secretion system gene *tsi2*, genes encoding the soluble bacteriocin pyocin S4, chemotaxis genes, and Psl polysaccharide synthesis gene *pslI*.

Alternatively, there were 80 genes required for survival of *P. aeruginosa* in physiologically-relevant *in vitro* media only, many of which belonged to functional classes that were also predicted as essential *in vivo*, e.g., those influencing energy metabolism, amino acid and nucleotide metabolism, membrane integrity, and iron acquisition (Supplementary Table S5; Figure 1B). It is likely these functional classes are not discriminatory and are required for both *in vitro* and *in vivo*/*in vivo*-like conditions, but any differences might reflect differences in the wiring of metabolic pathways.

These data supported that genes identified in physiologically-relevant *in vitro* media provide a good representation of conditionally important genes in the host but likely did not provide an exhaustive list of all essential functions, and therefore has limitations.

### Genes shared between all physiologically-relevant conditions represent a robust example of genes required to survive in physiologically-relevant media

To determine trends in *P. aeruginosa* gene requirements in the utilized infection models, we compared conditionally required genes identified *in vitro,* in murine abscess, and in human skin models and found 110 shared genes between the three conditions when excluding genes essential in MHB (Figure 1; Supplementary Dataset 2). Classification of these 110 genes into Pseudocap functional classes identified membrane-related functions (14 genes), amino acid metabolism (8), biosynthesis of cofactors and prosthetic groups (4), nucleotide biosynthesis and metabolism (6), transcriptional regulation (6), and transport of small molecules (11) as the most represented (Supplementary Dataset 2). Genes in these functional classes were explored in more detail below.

### Tn-Seq identified virulence and membrane transport genes previously implicated as important for survival in the host environment

Previous studies examining genes important for survival of *P. aeruginosa* grown under physiologically relevant growth conditions have indicated that virulence, secretion and iron acquisition are important factors involved in survival and infection (Reimmann et al., 2001; Galle et al., 2012; Turner et al., 2014; Lee et al., 2015; Turner et al., 2015; Poulsen et al., 2019). We found that several genes encoding proteins involved in transport, secretion and membrane integrity were predicted as essential in all physiologically-relevant conditions in this study (Table 2). This included genes important for transport of divalent cations and metallic cations such as Fe^3+^, e.g., adjacent genes encoding the pyochelin synthesis proteins, PchG and PchF (Supplementary Dataset 2). Identifying genes involved in iron uptake is noteworthy, since *P. aeruginosa* has multiple systems for synthesizing siderophores, which are be diffusible molecules that bind and uptake iron. For this reason, we would expect the mutants to be complemented in trans. However, in our models, we found pyochelin synthesis mutants in the Tn-Seq pools did not survive. This being said, iron acquisition genes have also been identified in previous Tn-Seq studies in murine chronic wound models (Supplementary Table S6; Turner et al., 2014) and it is well known that they play an important role in infection and virulence.

**Table 2:** Selected *Pseudomonas aeruginosa* PAO1 genes belonging to pathways essential under either RPMI or RPMI/serum as well as in murine abscess and human skin models.

Gene	Description	Essential in			
		RPMI/Serum	RPMI	Abscess	Skin
Amino acid metabolism and biosynthesis					
*aceE*	pyruvate dehydrogenase	ES^2^	ES^2^	ES^1^	ES^2^
*dapA*	dihydrodipicolinate synthase	ES	ES	ES	ES
*dchB*	dehydrocarnitine CoA transferase, subunit B	-	ES	ES	ES
*hisF2*	imidazoleglycerol-phosphate synthase subunit	-	ES	ES	ES
*masA*	enolase-phosphatase E-1	ES	ES	ES	ES
*nagZ*	beta-N-acetyl-D-glucosaminidase	ES	ES	ES	ES
*pheA*	chorismate mutase	-	ES	ES	ES
Biosynthesis of cofactors, prosthetic groups and carriers					
*cobC*	cobalamin biosynthetic protein	-	ES	-	ES
*cobD*	cobalamin biosynthetic protein	ES^2^	ES	ES	ES
*cobE*	cobalamin biosynthetic protein	-	-	-	ES
*cobH*	precorrin isomerase	ES	-	-	-
*cobO*	cobalamin adenosyltransferase	-	ES	ES	-
*cobQ*	cobyric acid synthase	-	ES	ES	ES
*pqqF*	pyrroloquinoline quinone biosynthesis protein F	-	ES	ES	-
*pqqD*	pyrroloquinoline quinone biosynthesis protein D	ES	ES	ES	ES
Membrane/transport small molecules					
*ampO*	Iron regulated membrane componenet	-	ES	ES	ES
*copA2*	copper transport ATPase	ES^1^	ES^1^	ES^2^	ES^2^
*fpvF*	ABC transporter involved in iron uptake	-	ES	ES	ES
*pchG*	pyochelin biosynthetic protein	-	ES	ES	ES
*pchF*	pyochelin synthetase	ES^1^	ES^1^	ES^1^	ES^1^
*shaD*	sodium:Hydrogen antiporter	-	ES	ES	ES
*shaF*	sodium:hydrogen antiporter	ES	ES	ES	ES
*napF*	Ferredoxin protein	ES	ES	ES	ES
PA2297	Probable ferredoxin	-	ES	ES	ES
*napE*	Periplasmic nitrate reductase protein	-	ES	ES	ES
Nucleotide biosynthesis and metabolism					
*carA*	carbamoyl-phosphate synthase small chain	ES^1^	ES^1^	ES	ES
*dnaQ*	DNA polymerase III, epsilon chain	-	ES	ES	ES
*purD*	phosphoribosylamine-glycine ligase	ES^1^	ES^2^	ES	-
*purE*	phosphoribosylaminoimidazole carboxylase subunit	ES	ES	ES	ES
*purF*	amidophosphoribosyltransferase	ES^2^	ES^2^	ES^2^	-
*purH*	phosphoribosylaminoimidazolecarboxamide formyltransferase	ES^2^	ES^2^	ES^2^	-
*pyrC*	dihydroorotase	ES^2^	ES	ES	-
*pyrD*	dihydroorotate dehydrogenase	ES	-	ES	ES
*pyrF*	orotidine 5′-phosphate decarboxylase	ES	ES	ES	ES
PA3505	Nicotinate and nicotinamide metabolism	ES	ES	-	ES
*sth*	soluble pyridine nucleotide transhydrogenase	-	ES^2^	ES	ES
Protein secretion and export					
*pscB*	type III export apparatus protein	ES	ES	ES	ES
*pscO*	translocation protein in type III secretion	-	ES	ES	ES
*tatB*	translocation protein	-	ES	ES	ES
*tatA*	translocation protein	-	ES	ES	ES
*hxcU*	Pseudopillin; alk. Phosphatase secretion	-	-	ES	ES
*hxcW*	Pseudopillin; alk. Phosphatase secretion	-	ES	-	-

Essential by Tradis = ES; Transit = ES^1^; Both methods = ES^2^; Neither method (i.e., not found to be essential) = −.

Other genes involved in membrane functions and transport of small molecules, that were predicted as essential in host mimicking conditions in this study (Table 2; Supplementary Table S6) as well as previous studies included (a) genes encoding ferredoxin protein PA2297 and nitrate reductase NapF/NapE, which were also found to be essential in murine wound models (Turner et al., 2014), (b) sodium/hydrogen antiporter proteins (ShaDF) implicated as important in sodium homeostasis and virulence of *P. aeruginosa in vivo* (Kosono et al., 2005) and previously found to be essential in murine wounds, SCFM and lung sputum (Turner et al., 2014, 2015; Lee et al., 2015), (c) copper transport gene *cop*A2 which was previously found to be essential in wound, bovine serum, and SCFM (Turner et al., 2014, 2015; Poulsen et al., 2019), as well as (d) translocase genes coding for the TatABC and Psc operons and HxcUW pseudopilins important for type 2 (T2SS) and type 3 (T3SS) secretion systems and previously implicated as essential in wound, bovine serum, and SCFM models (Turner et al., 2014, 2015; Morgan et al., 2019; Poulsen et al., 2019).

Since many virulence and transport genes had overlapping essentiality between this study and previous studies, we examined whether these genes also had similar transcriptional patterns in RPMI/serum to those observed in studies exploring physiologically-relevant conditions. To do this, previously published RNA-Seq data collected from *P. aeruginosa* grown under RPMI/serum *cf.* MHB (Belanger et al., 2020) were compared to qRT-PCR analysis in the murine abscess model, (Pletzer et al., 2017b), and transcriptomic data in chronic and acute murine wound infections (Supplementary Table S7; Turner et al., 2014). Both previous abscess/wound studies focused on pyoverdine and phenazine iron uptake pathways, regulation of T3SS and T2SS, alginate synthesis, Psl polysaccharide production, motility, and rhamnolipid production as markers for virulence *in vivo*. Transcriptomics of *P. aeruginosa* grown *in vitro* in RPMI/serum *cf.* MHB (Belanger et al., 2020) revealed increased expression of selected pyoverdine genes, and T2SS and T3SS genes, as was observed *in vivo,* but no alteration of expression of other genes involved in motility and rhamnolipid synthesis (Supplementary Table S7). This comparison implies similar regulatory patterns in secretion systems and virulence factors between host like conditions, but differences in the behavior of specific genes involved in motility and attachment. By observing essentiality and expression patterns of genes involved in virulence in both *in vitro* and *in vivo* host mimicking conditions compared to MHB, we found that some of the genes identified in this study had also been identified by previous studies (Lee et al., 2014; Turner et al., 2014, 2015; Pletzer et al., 2017b; Poulsen et al., 2019) in different host environments. These observations support that the host mimicking conditions used here, reflect a sufficient representation of genes considered essential for infection of *P. aeruginosa* in the host environment.

### Genes involved in microbial metabolic functions were required for growth under physiologically-relevant conditions

In addition to virulence factors and membrane transport genes, we found that a large proportion of classified essential genes in host mimicking conditions belonged to pathways involved in microbial metabolic functions. These genes were of interest since they might indicate altered metabolism and potential metabolic targets of *P. aeruginosa* in infection environments.

Amino acid metabolism genes that were required for survival in both abscess and skin models as well as physiologically-relevant *in vitro* media included genes encoding proteins for phenylalanine, tyrosine, lysine, histidine and methionine biosynthesis and utilization (Table 2; Klem and Davisson, 1993; Myers et al., 1993; Xie et al., 1999). The genes *carA* that encodes a subunit of carbamoyl-phosphate, involved in catalyzing the biosynthesis of a precursor for arginine and pyrimidines (Cunin et al., 1986), and *nagZ* that codes for a beta-N-acetyl-D-glucosaminidase involved in β-lactam resistance (Zamorano et al., 2010) were also required.

In this study, we also found that genes *pyrF*, *purE*, and *carA* belonging to pyrimidine and purine metabolism operons (Supplementary Figure S2), were required for survival in all media conditions (Table 2). Additionally, *pyrD*, *purF*, *purH*, and hypothetical pyrimidine biosynthesis gene PA3505 were predicted essential in 3 out of 4 host-like conditions. Genes belonging to purine and pyrimidine metabolism have been previously suggested to play an essential role in *P. aeruginosa* survival in host environments (Supplementary Table S6) such as blood (Samant et al., 2008), human serum (Weber et al., 2020), *in vivo* infection models (Turner et al., 2014), and in bovine serum (Poulsen et al., 2019) and SCFM (Turner et al., 2015).

Genes belonging to operons involved in carbon catabolism and biosynthesis of cofactors had interesting overlaps between *in vivo* and *in vitro* host mimicking conditions. Malonate decarboxylase genes *mdcC* and *mdcE* were required for survival in all physiologically-relevant conditions (Table 2) and were also previously identified as important in murine wound models (Turner et al., 2014). Genes involved in the biosynthesis of pyrroloquinoline quinone (PQQ) and cobalamin (Cob; Supplementary Figure S3) were required in some or all physiologically-relevant conditions. PQQ is a co-factor involved in energy metabolism and previously demonstrated to be induced during growth on ethanol, 1-propanol, 1,2-propanediol and 1-butanol (Gliese et al., 2010). Cobalamin, or vitamin B_12_, is a complex cofactor containing a central chelated cobalt ion. *CobC, cobD, cobO* and *cobQ* were required in all physiologically-relevant conditions, and others such as *cobE* and *cobH* were essential in only certain physiologically-relevant media. Similar to iron acquisition, it was surprising to identify cobalamin biosynthesis as essential, since one would expect that loss of cobalamin biosynthesis would be complemented by other mutants in the pool. Nevertheless, the levels of cobalamin in human serum and RPMI are very low when compared to other vitamins (Supplementary Table S8), and this pathway has also been demonstrated as essential in other *in vivo* models (Turner et al., 2014), which could make it a limiting nutrient for survival in these conditions.

### Integration of essential *Pseudomonas aeruginosa* pathways involved in nucleotide metabolism and cobalamin synthesis with gene expression in RPMI/serum show similar patterns

The availability of RNA-Seq data for *P. aeruginosa* grown under *in vitro* physiologically relevant conditions of RPMI and RPMI/serum (Belanger et al., 2020) gave us the ability to compare patterns of significantly enriched gene expression in these conditions, to the Tn-Seq data collected in this study. It was hypothesized that there would be overlapping enrichment in gene expression of pathways that were essential for survival of *P. aeruginosa* in the host environment. Conditionally essential genes in RPMI and/or RPMI/serum were integrated with RNA-Seq from *P. aeruginosa* grown in RPMI (Belanger et al., 2020), using a new web tool, PaIntDB, (Castillo-Arnemann et al., 2021) that maps such data to protein–protein interaction (PPI) networks in *P. aeruginosa*. PPI networks comprised nodes (circles) representing gene-encoded proteins connected by lines/edges that represent known or extrapolated PPIs representing physical, metabolic or regulatory interactions. Using the RNA-Seq and essential Tn-Seq genes, a zero-order PPI network of 1,921 genes/proteins was constructed, involving only direct interactions between these nodes., including 1,856 nodes from RNA-Seq data and 175 from Tn-Seq data. Using the ontologies tool in PaIntDB, subnetworks were constructed for pathways that were significantly enriched in these datasets. There were a total of 150 significantly enriched gene ontology (GO) terms found (*p* < 0.05).

**Figure 2:**
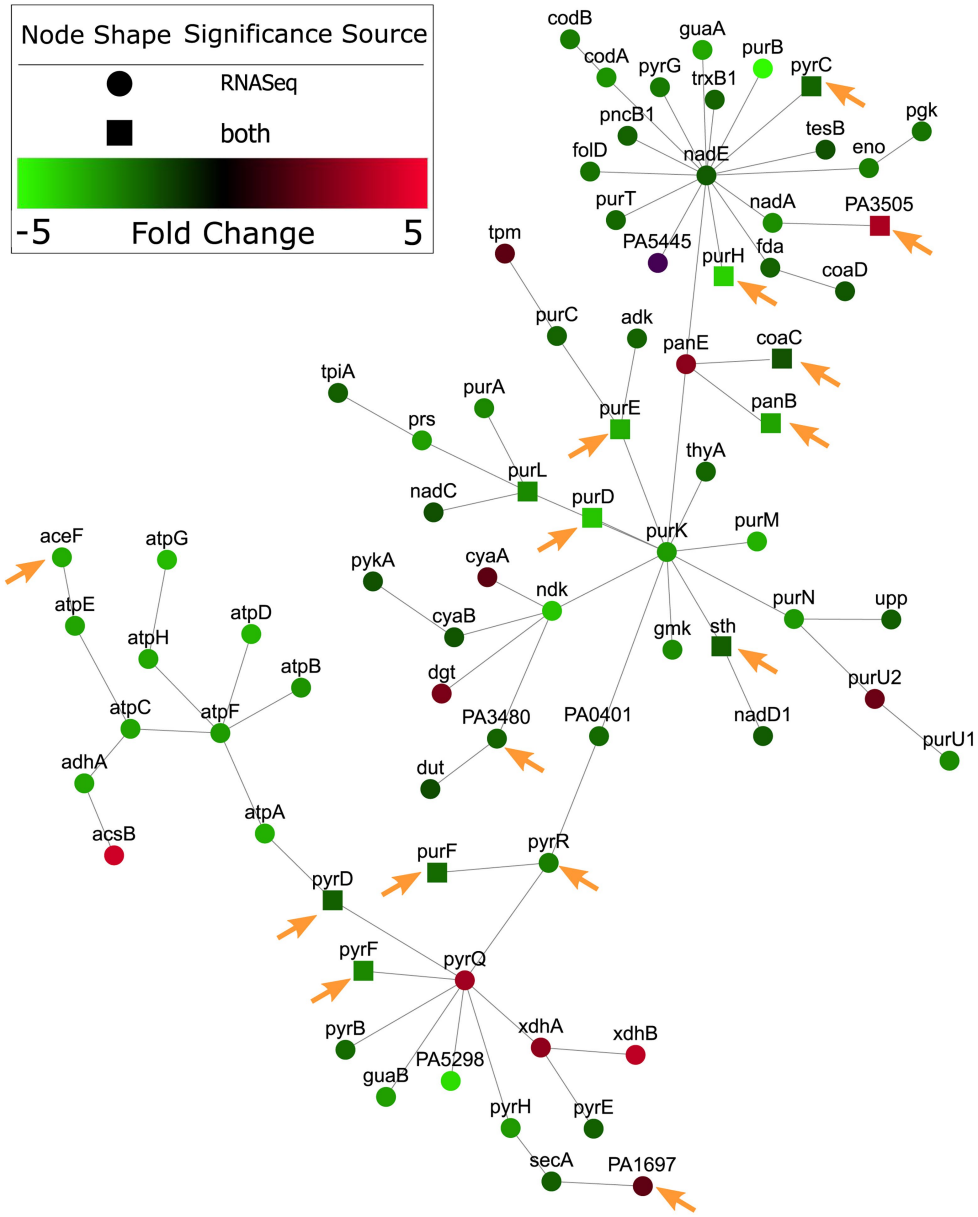
Nucleotide metabolism genes were predicted as conditionally essential, enriched and differentially expressed in RPMI *cf.* MHB. Conditionally essential genes in RPMI and/or RPMI/serum were integrated with RNA-Seq from *P. aeruginosa* grown in RPMI, and mapped to PPI networks in *P. aeruginosa*. A network of enriched gene ontology (GO) terms for nucleotide metabolism and biosynthesis is visualized here. Genes indicated with a square were essential in RPMI and/or RPMI/serum but not MHB. Those genes that were also essential in abscesses *cf.* MHB and/or the human skin model *cf.* MHB are indicated with orange arrows.

**Figure 3:**
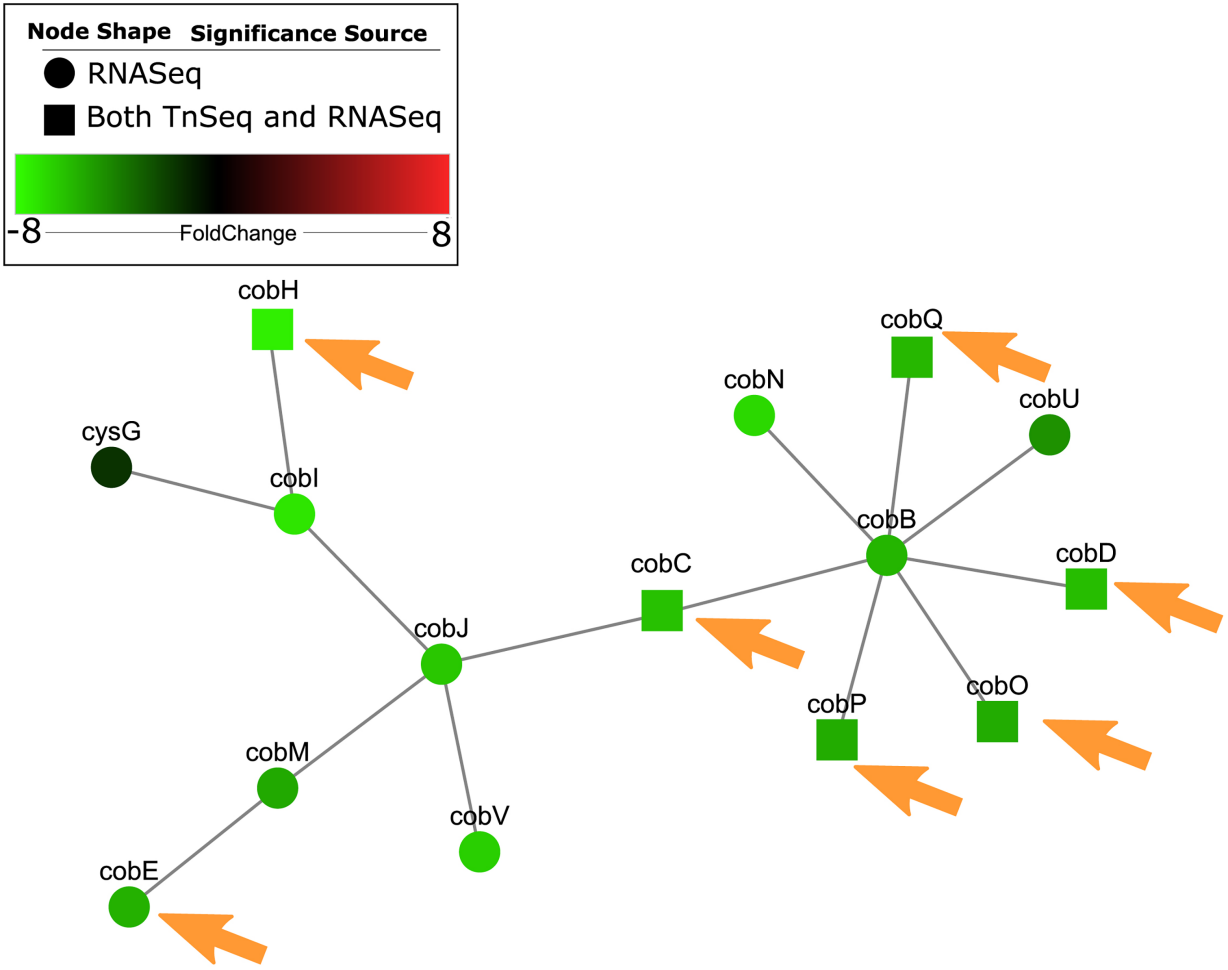
Cobalamin metabolism genes were predicted as conditionally essential, enriched and differentially expressed in RPMI *cf.* MHB and identified as conditionally essential for growth in physiologically-relevant conditions. Conditionally essential genes in RPMI and/or RPMI/serum were integrated with RNA-Seq from *P. aeruginosa* grown in RPMI *cf.* MHB, and mapped to PPI networks in *P. aeruginosa*. A network of enriched GO terms for cobalamin metabolism and biosynthesis is visualized here. Those genes that were also essential in abscesses *cf.* MHB and/or the human skin model *cf.* MHB are indicated with orange arrows.

A subnetwork was identified containing purine and pyrimidine byosinthesis and metabolism pathways with 76 enriched genes, 12 of which were conditionally essential in RPMI or RPMI/serum *cf.* MHB (Figure 2). The majority (10/12) of these essential genes were also observed to be essential in the murine abscess models, while five were essential in the skin model. Five genes were essential in both the abscess and skin models. Interestingly, most of the genes in these pathways were downregulated by up to 4-fold when compared to MHB, which was consistent with the findings of Turner et al. in murine chronic and acute wounds compared to MOPS-succinate (Turner et al., 2014). Although a smaller number of genes involved in biosynthesis of cofactors, prosthetic groups and carriers were indicated to be essential for survival *in vivo*, cobalamin biosynthesis was conditionally important and was also enriched in RNA-Seq data (Belanger et al., 2020) in host mimicking *in vitro* media (Figure 3). A subnetwork of 15 genes/proteins was obtained; of these, six were conditionally required in RPMI and/or RPMI/Serum, two in both abscess and skin, and one in only abscess or skin, respectively. Notably, *cobD, cobQ, cobO,* and *cobC* genes were essential for growth in multiple physiologically-relevant conditions tested, with *cobD* being found as essential in all conditions *cf.* MHB (Table 2). These genes were also downregulated under physiologically-relevant conditions when compared to MHB.

Therefore, integration of RNA-Seq and Tn-Seq data indicated the global importance of nucleotide and cobalamin synthesis pathways under physiological conditions, likely extenuated by the general downregulation of these pathways.

### Alternative nucleotide biosynthesis genes are upregulated in physiologically-relevant conditions

Since nucleotide metabolism was both essential and downregulated in physiologically relevant media, we hypothesized that a lack of precursor metabolites required for biosynthesis of nucleotides could explain the requirement and downregulated of genes from the nucleotide biosynthetic pathways under physiologically-relevant conditions when compared to MHB. We explored the available RNA-Seq data (Belanger et al., 2020) and found that genes required for the utilization of precursor metabolites for pyrimidines and purines were indeed upregulated (Supplementary Table S9). These included, the *arcC* (Baur et al., 1989) and *ansB* (Holcenberg et al., 1978) genes encoding elements of the arginine metabolic pathway catalyzing the production of carbamoyl phosphate from glutamine, which can then be used in pyrimidine metabolism, as well as glutamate catabolic genes *pauABCD* (Yao et al., 2011), and histidine catabolic genes *hutGHIU* which are involved in the production, from proteins, of amino acids previously proposed to be available in the host environment (Turner et al., 2014). Interestingly, genes involved in histidine metabolism were also essential under physiologically-relevant conditions, and it is possible that increased catabolism of arginine and histidine could be shuttled into increasing glutamine production, which could then be used in purine and pyrimidine metabolism. Similarly, an alternative gene proposed to be involved in pyrimidine synthesis, *panE,* was also upregulated under these conditions, providing a potential alternative path to pyrimidine synthesis in the host environment. These data are thus consistent with the importance of nucleotide biosynthesis *in vivo* and support that alternative pathways for nucleotide metabolism in the host environment might be utilized due to nutrient limitations in this environment.

### Confirmation of growth defects in mutants of genes involved in nucleobase and cobalamin biosynthesis in host mimicking *in vitro* media and *in vivo*

**Figure 4:**
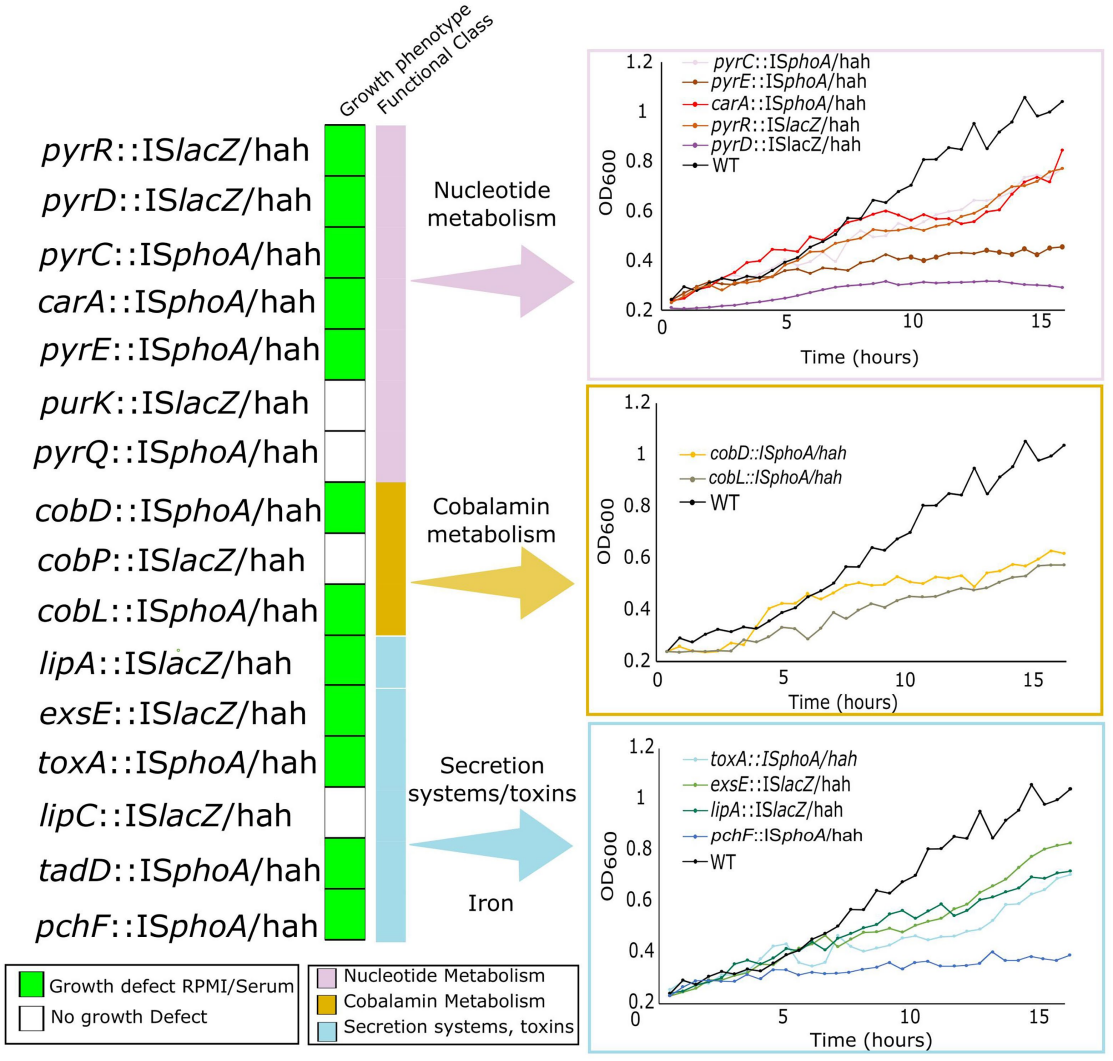
Growth defects of Tn-insertion mutants from the ordered PAO1 Tn mutant library in genes identified as important for survival in physiologically-relevant media and in murine abscess or the human skin model. Mutants harbouring Tn5 IS50L derivative Tn insertions IS*lacZ*/hah or IS*phoA*/hah (indicated as Tn5IS*lacZ* or Tn5IS*phoA*) were grown in both RPMI/Serum and MHB for 16 h in 96-well plates and OD_600_ was measured every 30 min. Growth curves in RPMI/serum for mutants that showed a growth defect in RPMI/serum but not in MHB (indicated by the green boxes on the left) are shown on the right and compared to WT PAO1 grown in the same media.

To validate growth defects of selected essential genes belonging to the same pathways as genes identified as conditionally essential, we utilized ordered Tn-insertion mutants from the PAO1 library (Liberati et al., 2006). We selected all mutants for genes in nucleotide metabolism, and cobalamin biosynthesis that were found in the ordered Tn library. These included *pyrC*, *pyrD*, *pyrE*, *pyrQ, pyrR*, *purK*, and *carA* for nucleotide metabolism, and *cobD*, *cobL,* and *cobP* for cobalamin metabolism. In addition, iron acquisition gene *pchF* and secretion/toxin system genes *exsE, toxA,* and *lipA* were included to validate the importance of iron acquisition and virulence. Mutants were first tested for growth in physiologically-relevant *in vitro* media, *cf.* MHB in a 96 well microtitre growth set up (Figure 4). Growth defects in RPMI/serum were observed in 12 out of 16 mutants for genes involved in toxin secretion, and metabolism of nucleobases, vitamin B_12_, and iron, with the largest defects observed for *pyrD*, *pyrE*, *tadD*, *cobD*, *cobL* and *pchF*. The remaining nine mutants had no growth defects in either medium. The lack of a growth defect despite conditional essentiality might be due to a competitive disadvantage in the Tn-Seq studies, *cf.* other mutants in the pool, that was not present when grown as isolated mutants.

**Figure 5:**
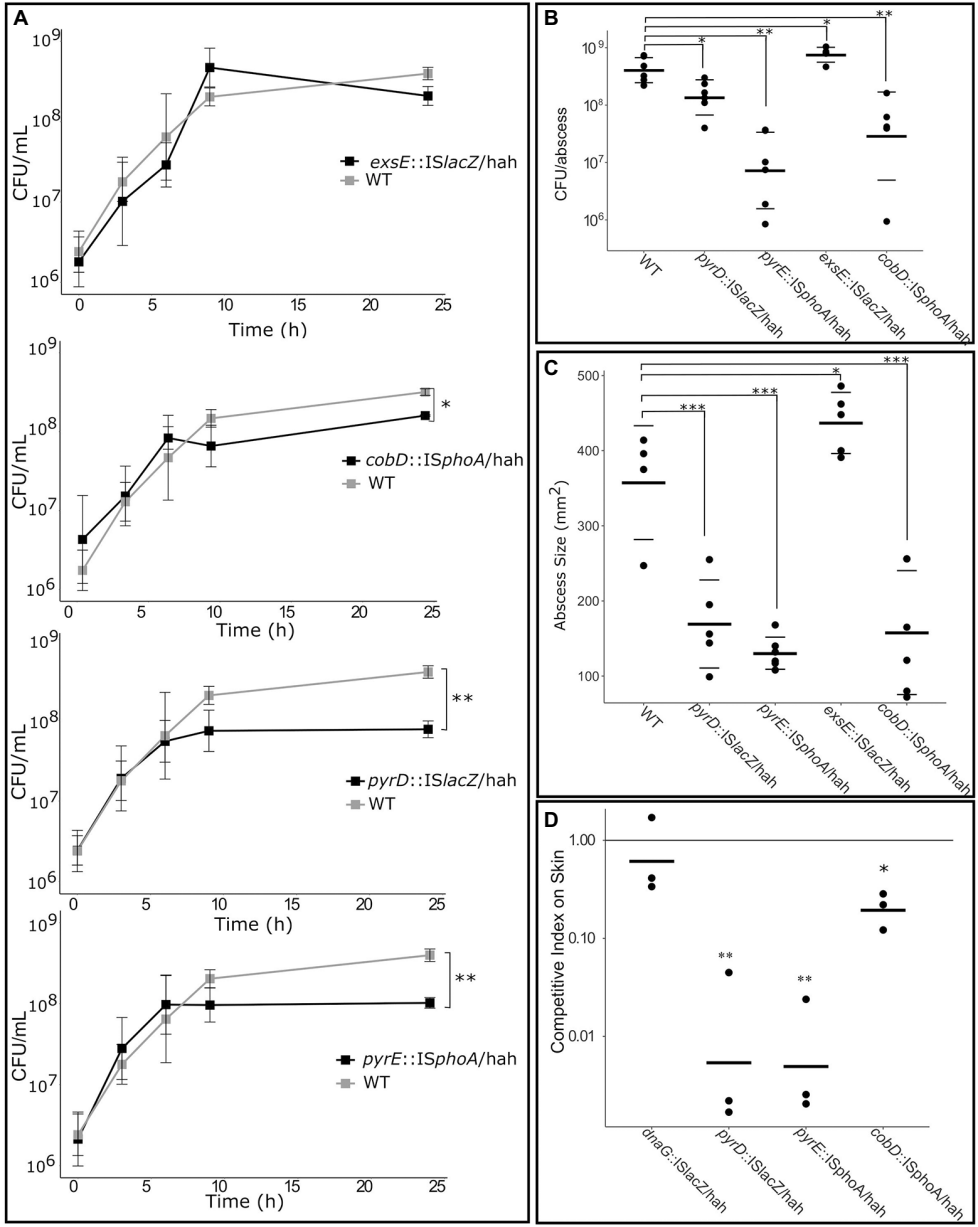
Deficiencies of mutants identified as important for growth in RPMI/Serum, murine abscess and/or a human skin model. **(A)** Growth defect compared to WT when grown in RPMI/Serum. *p < 0.05, **p < 0.01, ***p < 0.001 indicates significantly different from WT using two-way ANOVA. **(B)** Survival and **(C)** abscess formation in the murine abscess model. *p < 0.05, **p < 0.01, ***p < 0.001 indicates significantly different from WT using one-way ANOVA. **(D)** Competitive fitness of mutants in the human skin organoid model, *cf.* WT, measured as competitive index after inoculation with equal numbers of WT and mutant on the skin. *p < 0.05, **p < 0.001 indicated significantly different than 1 using 1-sample *t*-test.

Four mutants of interest were selected for more detailed assessment, including mutants for nucleobase metabolism genes *pyrD::lacZ* and *pyrE::phoA*, cobalamin metabolism gene *cobD::phoA*, and the negative regulator of T3SS *exsE::lacZ* as a virulence control. Growth kinetics were first investigated by inoculation of each of these mutants and WT PAO1 into media flasks at a starting concentration of 1 × 10^6^ CFU/ml. Cultures grown for 20 h at 37°C, and plated after 3, 6, 9 and 20 h to assess CFU/mL (Figure 5A). The *exsE* mutant showed no significant change in growth, while the *pyrD*, *pyrE* and *cobD* mutants had significant defects in growth in RPMI/serum, with 5.0 (*p* = 0.006), 3.9 (*p* = 0.005), and 1.7 (*p* = 0.02) fold less bacteria *cf.* WT, respectively.

Additionally, 2.5 × 10^7^ CFU of each mutant or WT were inoculated subdermally in mice to determine their ability to survive and form an abscess after 18 h (Figures 5B,C). The *exsE* mutant showed no growth defect and in fact demonstrated a slight but significant advantage in both CFU counts (2-fold more than WT, *p* = 0.03) and pathology (1.4-fold larger abscess than WT, p = 0.02). The *pyrD* and *pyrE* mutants demonstrated significantly decreased survival *in vivo* (2.8-fold, p = 0.02, and 29.4-fold, p = 0.005, less bacteria in abscesses *cf.* WT, respectively) as well as pathology, based on their ability to form abscesses (1.7 and 2.3-fold smaller abscess than WT for the *pyrD* (*p* = 0.0001) and *pyrE* (*p* = 7 × 10^−5^), mutants, respectively). Despite the fact that the *cobD* insertional mutant had only slightly decreased growth *in vitro* when compared to WT, in the murine model it exhibited 2.1-fold (*p* = 0.0008) and 7.9-fold (p = 0.006) decreases in abscess size and bacterial counts, respectively. To determine the effects that these mutations had on survival in the human skin organoid model, Tn mutants were mixed with WT PAO1 at a starting ratio of 1:1 and inoculated onto developed skin. The ratio of mutant to WT bacteria was used to assess the competitive index (CI) of the mutants after 48 h incubation on the skin (Figure 5D). A CI lower than one indicated a relative competitive fitness defect for that organism. As a negative control, we utilized a *dnaG::*IS*lacZ*/hah Tn mutant with an insertion at the end of this gene that did not disrupt the activity of this generally essential gene; it demonstrated no effect on growth rate in the skin organoid model with a CI of 0.82, that was not significantly different from WT (using a 1-sided t-test). The two mutants for nucleobase metabolism genes had the greatest growth defect in the human skin organoid model with CIs of 0.02 for *pyrD::lacZ* (*p* = 0.0002) and 0.01 for *pyrE::phoA* (*p* = 0.00005). The *cobD* mutant was also significantly reduced in growth in the skin model compared to WT with a CI of 0.21 (*p* = 0.004).

## Discussion

This study contributes to our understanding of genetic functions in *P. aeruginosa* that are needed to survive in physiologically-relevant *in vitro* media, in a murine chronic wound model, and in human skin infections. Exploration of these essential functions in *P. aeruginosa* was achieved by first generating a saturated Tn-Seq pool in PAO1 to enable comparison of genes conditionally important for survival *in vitro* and *in vivo*. Sequencing of the T0 Tn-Seq pool predicted 654 essential genes in *P. aeruginosa* when combining the two analysis tools Transit and Tradis. This number was larger than those found in three of six published datasets on *P. aeruginosa* (Lee et al., 2015; Turner et al., 2015), but was similar to that for three other studies (Liberati et al., 2006; Skurnik et al., 2013; Poulsen et al., 2019) examining essentiality of *P. aeruginosa* under different conditions (Table 1). Despite this study being performed in different media and using a different strain, and different analysis tools than some previous studies, all essential gene lists in this study and in previous research were considered to have significant overlap (Supplementary Dataset 1) and were composed of genes with similar overall functional properties (Supplementary Table S2). These data suggest that essential genes in *P. aeruginosa* are primarily involved in core metabolism, transcription and translation, and cell integrity, and that the analysis method used in this study is a robust predictor of classes of genes important for survival.

Genes conditionally important in physiologically-relevant media RPMI and/or RPMI/serum, in the murine abscess *in vivo* model, and in the human skin organoid model (Figure 1), were predicted by identifying all surviving mutants of the Tn pool in each of these conditions, compared to that obtained in nutrient rich laboratory medium MHB, commonly used for measuring antimicrobial susceptibility (Wiegand et al., 2008). A key finding from this research was that genes conditionally required for survival in host mimicking *in vitro* conditions and *in vivo* were significantly overlapping, and that many of these genes were also identified in previous studies (Table 2; Supplementary Table S6). A total of 110 genes were predicted to be important for survival under all of our physiologically-relevant conditions, but were not essential in MHB (Figure 1). This number represented approximately one third of the genes uniquely important in each of the skin model and murine abscess model, when compared to MHB, and almost half of the genes uniquely important for survival in physiologically-relevant *in vitro* media. The commonly important genes included those encoding proteins involved in membrane integrity, transport of small molecules, secretion, amino acid and nucleotide metabolism, and synthesis of cofactors. Remarkably, many of the genes in these functional classes that were implicated as important for *in vivo* survival, have also been directly or indirectly implicated as being important for antibiotic resistance or susceptibility in rich medium (Poole, 2007; Breidenstein et al., 2008; Kong et al., 2010; Zamorano et al., 2010), and have also been implicated in previous studies performed on both laboratory and clinical strains in other host environments (Turner et al., 2014; Poulsen et al., 2019). This observation supports the idea that the altered antimicrobial susceptibility that bacteria exhibit under *in vitro* host mimicking conditions (Belanger et al., 2020) and that observed in humans (Cornforth et al., 2018) is somehow linked, with a common requirement for certain functional pathways and genes for survival under these conditions.

Virulence and transport genes predicted to be essential *in vivo* were dysregulated during growth in the murine abscess model (Pletzer et al., 2017b), and we found that secretion systems, iron acquisition genes and toxins were similarly upregulated between *in vivo* and host mimicking *in vitro* conditions by comparison with previously published studies (Supplementary Table S7). Furthermore, Turner et al. (2014) demonstrated that genes involved in amino acid metabolism, transport of organic ions, and energy conversion and production were also significantly dysregulated in acute and chronic wounds when compared to minimal MOPS-succinate media. The alterations in gene expression under the host mimicking conditions utilized here shared the greatest similarity with chronic wound RNA-Seq data (Supplementary Table S7). Additionally, Tn-Seq predicted that genes involved in T3SS were important for survival in RPMI/Serum, abscesses, and the human skin model (Table 2). Consistent with this, the importance of the T2SS and T3SS in virulence *in vivo* has been well demonstrated in *P. aeruginosa* (Ball et al., 2002; Pletzer et al., 2017b). Intriguingly, an insertional inactivation mutant for the negative regulator of T3SS, *exsE*, (Rietsch et al., 2005) did not result in significantly altered growth rates in RPMI/serum (Figure 4) but led to increased virulence and abscess size in the murine abscess model (Figure 5). This supports the conclusion that the regulation of T3SS is essential for prolonged survival/virulence of *P. aeruginosa* during infection, and that a complete lack of negative regulation by ExsE can result in extremely virulent infections.

Microbial metabolic pathways engaged in the survival of *P. aeruginosa* in the host environment included metabolism of amino acids, nucleotides, and cofactors. The estimated concentrations of amino acids in blood plasma and serum differs widely depending on the study, the diet of individuals, and the time at which samples are taken (Krebs, 1950; Fukuda et al., 1984; Corso et al., 2017). However, compared to nutrient rich MHB (composed of beef extract, casein, and starch), most if not all amino acids are severely lacking (Supplementary Table S8). This suggests that there are crucial differences in amino acid availability and utilization in host mimicking conditions *cf.* MHB that are of interest in comparisons to genes essential *in vivo*. Furthermore, it was previously proposed (Turner et al., 2014) that certain metabolites were poorly or not available in the host environment during bacterial wound infections, including the amino acids glutamine, tyrosine, phenylalanine, aspartic acid and asparagine, purine nucleotides and potentially pyrimidine nucleotides, lysine, and methionine. Indeed, we identified here that pathways involved in biosynthesis of most of the least-available amino acids *in vivo*, as well as purines, and pyrimidines were required for *P. aeruginosa* survival, both *in vivo* and under physiologically-relevant *in vitro* conditions *cf.* MHB.

The importance of purine and pyrimidine metabolism genes found here corroborates previous studies, which include those performed on *P. aeruginosa* grown in acute and chronic murine wounds (Turner et al., 2014) and in fetal bovine serum (Poulsen et al., 2019), in *K. pneumoniae* grown in human serum (Weber et al., 2020), and in *Escherichia coli* grown in human blood (Samant et al., 2008). *P. aeruginosa* is capable of *de novo* pyrimidine synthesis using genes in the Pyr and Car pathways (Supplementary Figure S2) and also contains homologs (PA4396-PA4399) of genes from the secondary pyrimidine pathway of *Salmonella* (Frodyma and Downs, 1998). Purine biosynthesis uses the same backbone as pyrimidines, (phosphoribosyl pyrophosphate; PRPP), and production of both pyrimidine and purine nucleotides is positively regulated by glutamine and negatively regulated by intermediates of arginine and nucleotide biosynthesis (O’Donovan and Neuhard, 1970).

Additionally, we demonstrated that synthesis of cobalamin, a cofactor that was previously indicated to be potentially available in the host (Turner et al., 2014), was conditionally important for survival in our *in vivo* and *in vivo*-like models and under physiologically-relevant *in vitro* medium conditions when compared to nutrient rich medium. Vitamin B_12_, or cobalamin, is important for enzymatic activities in bacterial cells, including transmethylation, methionine synthesis, ribonuclease reductase, and anaerobic ethanolamine, glycerol and propanediol fermentation (Rodionov et al., 2003). In *P. aeruginosa* certain ribonucleotide reductases that catalyze the formation of deoxyribonucleotides from ribonucleotides, also require a cobalamin cofactor (Crespo et al., 2018). *Pseudomonas* can aerobically synthesize cobalamin *de novo* from aminolaevulinic acid (ALA), threonine and dimethyl benzimidazole as precursors and is also predicted to be able to salvage it from cobinamide (Fang et al., 2017; Supplementary Figure S3). Analysis of previous Tn-Seq datasets identified that *cob* genes were also conditionally important for survival in murine wound models (Turner et al., 2014), but were not required in bovine serum (Poulsen et al., 2019) or sputum containing media (Lee et al., 2015; Turner et al., 2015; Supplementary Table S6).

By combining Tn-Seq data with previously published RNA-Seq data (Belanger et al., 2020), we observed that the enriched genes involved in nucleotide biosynthesis and cobalamin biosynthesis were significantly downregulated in RPMI/serum when compared to MHB (Figures 2, 3). This downregulation was also observed in murine wounds where nucleobase synthesis genes were previously considered to be important for fitness, and a *purF* mutant was deficient for virulence in acute infections (Turner et al., 2014). Intuitively, one would expect that genes that are important for survival under particular circumstances might also be upregulated in that condition; however, research comparing global expression and phenotypic data has repeatedly found that there is no consistent correlation either way (Turner et al., 2014; Evans, 2015; Cain et al., 2020). Although these pathways are important for survival in host conditions, the nutrient limiting environment of the host lacks precursors for nucleotide and cobalamin synthesis *via* the traditional routes (Jaishankar and Srivastava, 2017). Analysis of RNA-Seq data (Belanger et al., 2020) from *P. aeruginosa* grown in RPMI/serum *cf.* MHB, indicated that histidine and glutamine catabolism were upregulated, possibly in an effort to produce metabolites that could be utilized in nucleotide synthesis. Alternative nucleotide metabolism genes were conversely upregulated in RPMI/serum when compared to MHB (Supplementary Table S9). Downregulation of cobalamin synthesis pathways in RPMI/serum compared to MHB, could be in response to limitation of precursor metabolites, and downregulation of processes requiring enzymes that utilize cobalamin as a cofactor. This downregulation might also explain why there were no rescue phenotypes in the Tn-Seq libraries grown in these conditions. Nevertheless, in these nutrient limiting environments, production of nucleotides and cobalamin were essential functions, and secondary screening experiments validated the importance of these genes for growth in RPMI/serum, murine abscesses, and the human skin organoid model (Figures 4, 5).

This research demonstrates that in physiologically-relevant media containing human serum, *P. aeruginosa* demonstrates strongly overlapping gene requirements for survival when compared to *in vivo* infections. The medium also accurately reflects the *in vivo* dysregulation of genes involved in iron uptake, secretion systems and toxin production when compared to nutrient rich conditions. Pathways involved in amino acid and nucleotide biosynthesis were validated for their importance *in vivo,* and vitamin B_12_/cobalamin biosynthesis was demonstrated to be a conditionally important function for *P. aeruginosa* survival under physiologically-relevant conditions and *in vivo.* In addition to increasing our understand of pathogenesis and survival in *P. aeruginosa* infection, this study highlights opportunities for progress in therapeutic development. Overlapping essentiality between *in vivo* and host mimicking conditions supports the use of physiologically-relevant media as a robust screen for antimicrobials (Belanger and Hancock, 2021).

## Data availability statement

The data presented in the study are deposited in the Gene Expression Omnibus as a BioProject with the accession number: GSE214167.

## Ethics statement

The animal study was performed following Canadian Council on Animal Care (CCAC) guidelines and was reviewed and approved by The University of British Columbia Animal Care Committee [certificate number A14-0363].

## Author contributions

CB and RH conceived the study. Most of the experimental design, experimentation, data analysis, writing of the paper and editing was performed by CB. MD significantly contributed to experimental design and Tn-Seq library construction. Library prep and sequencing of Tn-Seq experiments was performed by CB, MD, and RF. Bioinformatic analysis of Tn-Seq results was performed with the assistance of TB, AL, and BD. NR, DP, CB, and MD performed murine experiments. NA, BW, CB, and MD performed human skin organoid model experiments. JC-A assisted with PaIntDB analysis. CH assisted with Tn-Seq project design. RH obtained funding for this study and contributed to the writing of the paper. All authors edited and approved the manuscript.

## Funding

We gratefully acknowledge funding to RH from the Canadian Institutes for Health Research grant FDN-154287. RH holds a UBC Killam Professorship. CB was funded by a Cystic Fibrosis Canada graduate fellowship award #498801. MD was supported by the Graduate Award Program of the Centre for Blood Reasearch, UBC.

## Conflict of interest

The authors declare that the research was conducted in the absence of any commercial or financial relationships that could be construed as a potential conflict of interest.

## Publisher’s note

All claims expressed in this article are solely those of the authors and do not necessarily represent those of their affiliated organizations, or those of the publisher, the editors and the reviewers. Any product that may be evaluated in this article, or claim that may be made by its manufacturer, is not guaranteed or endorsed by the publisher.
