# Supplementary material for: Surviving the host: Microbial metabolic genes required for growth of Pseudomonas aeruginosa in physiologically-relevant conditions
Source: Front Microbiol. 2022 Nov 25;13:1055512. doi: 10.3389/fmicb.2022.1055512 (PMC9732424; doi:10.3389/fmicb.2022.1055512)
Supplement: Supplementary file 3 [file Data_Sheet_3.docx]

# Supplementary Material

**Methods: Amplification of transposon-genome junctions of the TnSeq library in PAO1**

The first PCR step contained 10 × Taq buffer (Thermo Scientific), 0.9 µM transposon-specific primer, R1-TnM20, 1.9 µM mixed degenerate arbitrary primers A-D (0.475 µM of each), 2.5 mM MgCl_2_, 0.2 mM dNTPs, 4% DMSO, 100 ng genomic DNA, 1 µL Taq polymerase and nuclease free water to 50 µL final volume. Cycling parameters were as follows 94 ℃ for 45 seconds, followed by 15 cycles of 30 seconds at 94 ℃, 30 seconds 49 ℃ annealing with reduction of 1 ℃ per cycle, and 1 minute at 72 ℃, then 20 cycles of 30 seconds at 94 ℃, 30 seconds at 60 ℃ and 1 minute at 72 ℃ , with final extension of 3 minutes at 72 ℃. Replicate PCR products were pooled together and cleaned with Agencourt AMPure XP magnetic beads (Beckman Coulter) with a bead ratio of 0.8. Each pool was eluted in a total volume of 40 µL nuclease free TE buffer and concentration and purity was determined using NanoDrop 2000. The second round PCR contained 10 × Taq buffer, 0.4 µM Tn-specific primer R2-TnM20, 0.4 µM ARB2 primer targeting the common repeat sequence on the arbitrary primers from step 1, 2mM MgCl_2_, 0.2mM dNTP, 4% DMSO, 100 ng DNA from first round PCR, 1 µL Taq polymerase and nuclease free water to 50 µL. The cycling parameters were as follows: 45 seconds at 94 ℃, 10 cycles of 30 seconds at 94 ℃, 30 seconds at 60 ℃, and 1 minute at 72 ℃, then a final extension step at 72 ℃ for 5 minutes.

**Supplementary Figure 1. Essential genes *in vitro*, in physiologically-relevant media RPMI and RPMI/serum when compared to MHB**. Tn-Seq was performed using a *P. aeruginosa* PAO1 mutant pool grown in physiologically-relevant media and MHB. Genes determined to be essential in each condition were those predicted by either Transit or Tradis. **(A)** The Venn diagram shows the total number of essential genes under each *in vitro* growth condition. **(B)** The bar graph shows numbers of unique essential genes in RPMI and/or RPMI/serum cf. MHB belonging to each PseudoCap (Winsor et al., 2016) functional class.


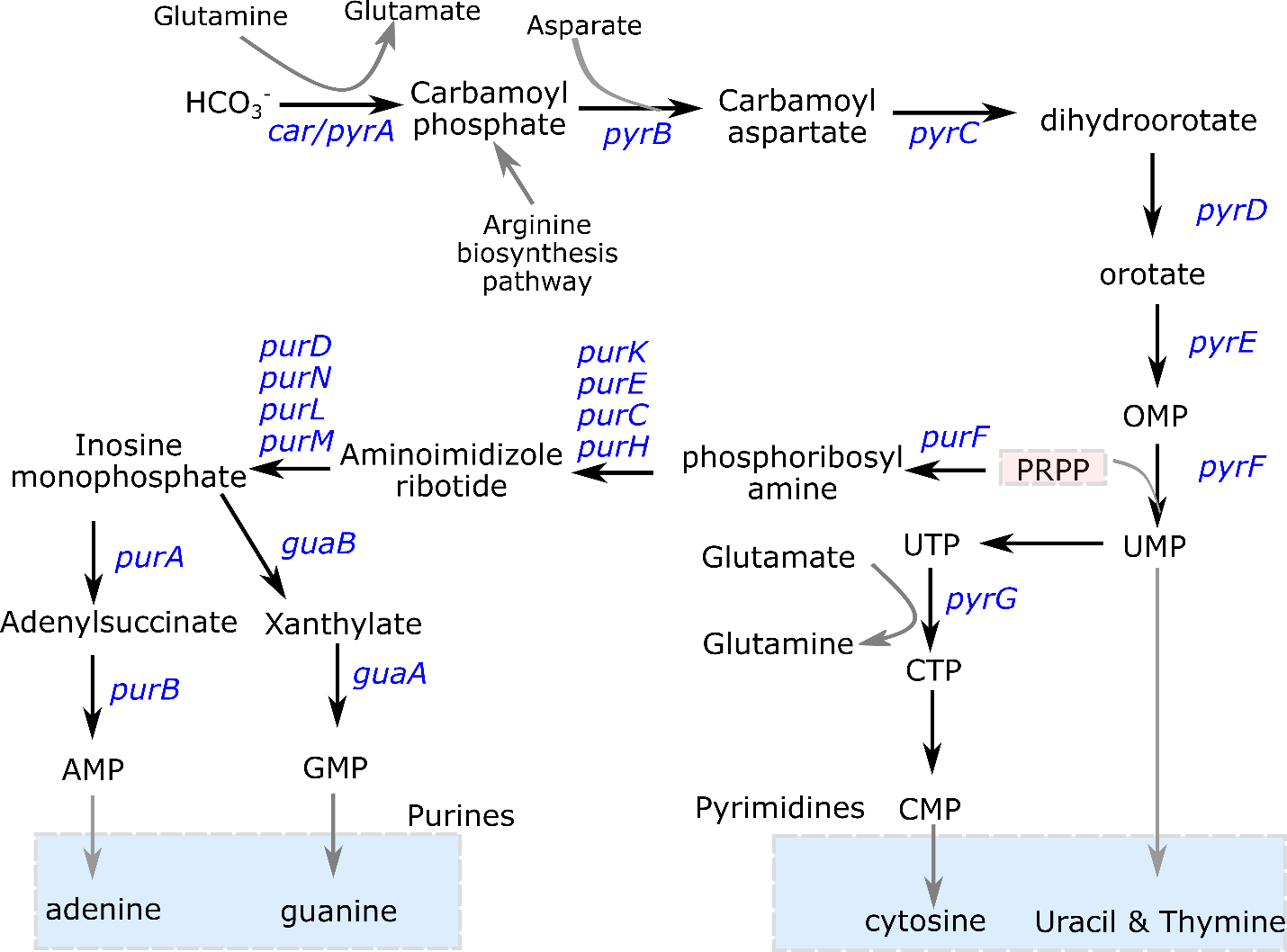


**Supplementary Figure 2. Purine and pyrimidine biosynthetic pathways.**


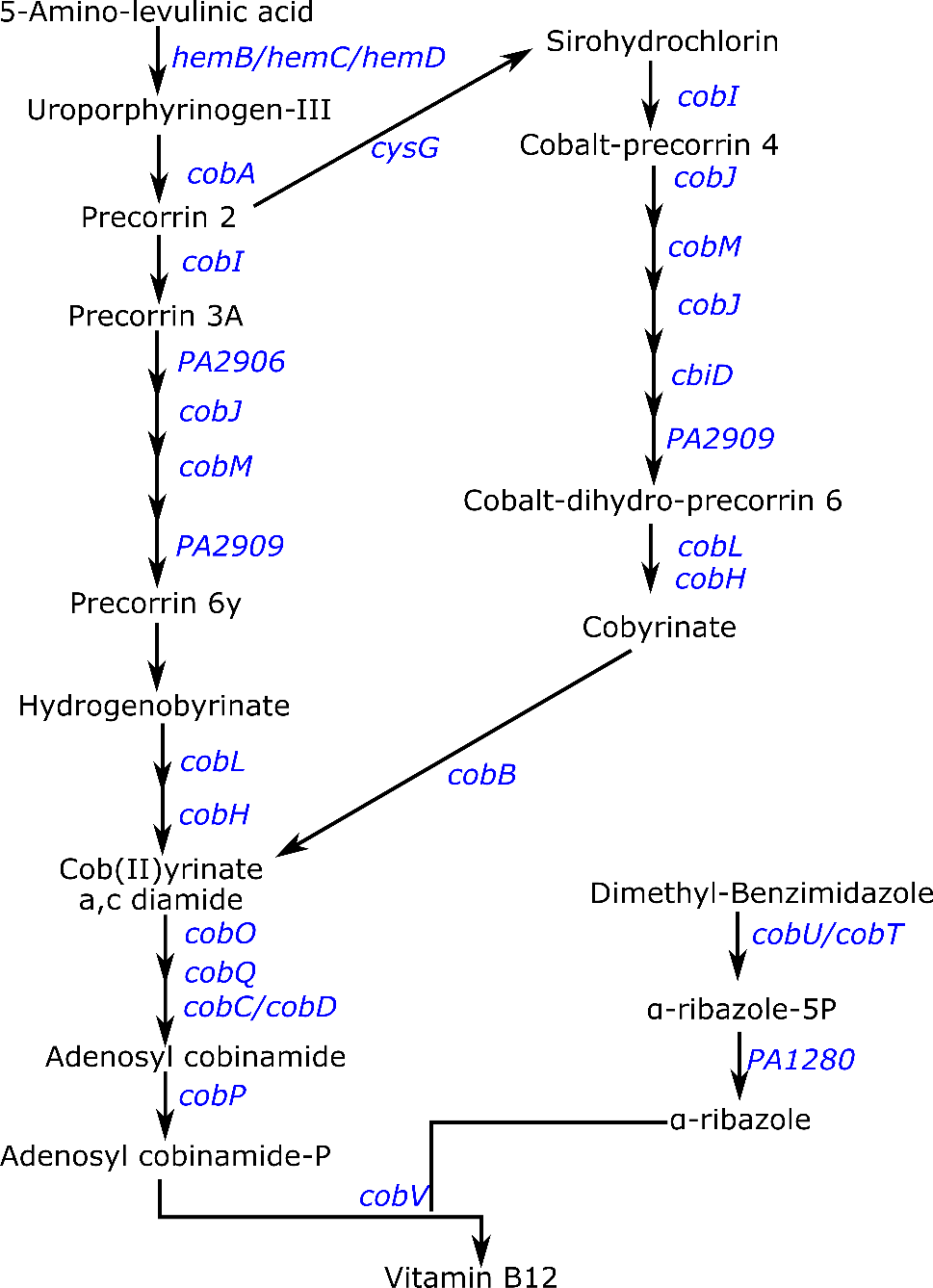


**Supplementary Figure 3. Cobalamin biosynthetic pathways**

**Supplementary Table 1. Primers used in this study.**

| **Name** | **Type** | **Sequence** |
| --- | --- | --- |
| R1-TnM20 | Tn-Seq; PCR1 | TATAATGTGTGGAATTGTGAGCGG |
| ARB1A | Tn-Seq; PCR1 | GCCACGCGTCGACTAGTACNNNNNNNNNNACGCC |
| ARB1B | Tn-Seq; PCR1 | GCCACGCGTCGACTAGTACNNNNNNNNNNTGCGG |
| ARB1C | Tn-Seq; PCR1 | GCCACGCGTCGACTAGTACNNNNNNNNNNTCCGG |
| ARB1D | Tn-Seq; PCR1 | GCCACGCGTCGACTAGTACNNNNNNNNNNGATAT |
| R2-TnM20 (TnM-P5-Seq) | Tn-Seq; PCR2 | TCGTCGGCAGCGTCAGATGTGTATAAGAGACAGCCGGGGACTTATCAGCCAACCT |
| ARB2(ARB2-Nex) | Tn-Seq; PCR2 | GTCTCGTGGGCTCGGAGATGTGTATAAGAGACAGGCCACGCGTCGACTAGTAC |

**Supplementary Table 2. Percentage of each functional class represented in the whole genome of *P. aeruginosa* PAO1 and in each rich media essential gene set analyzed in this study**. Statistically significantly enriched functional classes compared to the genomic reference set are bold (p < 0.05; hypergeometric distribution with FDR correction).

| Functional Class | % PAO1 genome | % Essential mutants in each Functional Class | | | | | | |
| --- | --- | --- | --- | --- | --- | --- | --- | --- |
|  |  | This study | Lee,et al. 2015 | Turner,et al. 2014 | Turner et al. 2015 | Skurnik et al. 2013 | Poulsen et al 2019 | Liberati et al. 2006 |
| Adaptation and protection | 3 | 2 | 2 | 2 | 1 | 2 | 2 | 2 |
| Amino acid metabolism and biosynthesis | 4 | **6** | **7** | **7** | **8** | 5 | **7** | 3 |
| Antibiotic resistance and susceptibility | 1 | 1 | 0 | 1 | 0 | 1 | 1 | 0 |
| Biosynthesis cofactors prosthetic groups and carriers | 2 | **7** | **9** | **8** | **9** | **4** | **9** | **4** |
| Carbon compound catabolism | 3 | 2 | 1 | 1 | 1 | 3 | 2 | 3 |
| Cell division | <1 | **2** | **3** | **2** | **3** | **2** | **3** | 1 |
| Cell wall LPS capsule | 3 | **7** | **10** | **7** | **12** | **5** | **8** | 3 |
| Central intermediary metabolism | 2 | 2 | 2 | 2 | 2 | 2 | 2 | 1 |
| Chaperones and heat shock proteins | 1 | 2 | 2 | 1 | 1 | 1 | 2 | 1 |
| Chemotaxis | 1 | 0 | 0 | 0 | 0 | 0 | 0 | 1 |
| DNA replication and repair | 1 | **3** | **4** | **6** | **4** | **4** | **4** | **2** |
| Energy metabolism | 3 | **5** | **7** | **7** | **7** | **7** | **6** | 4 |
| FA and phospholipid metabolism | 1 | **3** | **3** | **3** | **3** | 2 | **3** | 1 |
| Hypothetical unclassified | 29 | 18 | 11 | 13 | 9 | 19 | 11 | 32 |
| Membrane | 10 | 6 | 4 | 5 | 4 | 6 | 4 | 7 |
| Motility | 2 | 0 | 0 | 0 | 0 | 1 | 1 | 1 |
| Nucleotide biosynthesis and metabolism | 1 | **3** | **3** | **4** | **4** | **3** | **4** | 2 |
| Protein secretion and export | 2 | 3 | 2 | 2 | 2 | **4** | 2 | 3 |
| Putative enzyme | 7 | 4 | 3 | 2 | 4 | 4 | 4 | 5 |
| Related to phage | 1 | 0 | 0 | 0 | 0 | 0 | 0 | 1 |
| Secreted factors | 2 | 1 | 0 | 0 | 0 | 0 | 0 | 0 |
| Transcription RNA processing and degradation | 1 | **2** | **3** | **3** | **3** | **2** | **3** | 1 |
| Transcriptional regulator | 7 | 3 | 2 | 2 | 2 | 3 | 3 | 8 |
| Translation and post translational modification | 3 | **13** | **17** | **18** | **15** | **13** | **15** | **7** |
| Transport small molecules | 9 | 4 | 3 | 4 | 3 | 6 | 3 | 5 |
| Two component regulator | 2 | 1 | 0 | 0 | 0 | 1 | 0 | 1 |

**Supplementary Table 3. *P. aeruginosa* PAO1 genes predicted as essential in RPMI as well as RPMI/serum cf. MHB. Functional Classes** are: AA - Amino acid metabolism and biosynthesis; AN - Antibiotic resistance and susceptibility; AP - Adaptation and protection; BC - Biosynthesis, cofactors, prosthetic groups and carriers; CC - Carbon compound catabolism; CT - Chemotaxis; CM - Central intermediary metabolism; CD - Cell division; CW - Cell wall, LPS, capsule; CH - Chaperones and heat shock proteins; DN - DNA replication and repair; EM – Energy metabolism; EN - Putative enzyme; FA - Fatty acid and phospholipid metabolism; HY - Hypothetical unclassified; ME - Membrane; MO - Motility; NU - Nucleotide biosynthesis and metabolism; PH - Related to phage; RE - Transcriptional regulator; SE - Protein secretion and export; SF - Secreted factors; TC - Two component regulator; TL - Translation and post translational modification; TR - Transcription, RNA processing and degradation; TS - Transport small molecules.

| **Locus Tag** | **Gene** | **Essential in** | | **Functional Class** |
| --- | --- | --- | --- | --- |
|  |  | **Source RPMI/Serum** | **Source RPMI** |  |
| PA0002 | *dnaN* | Both | Tradis | DN |
| PA0187 | PA0187 | Tradis | Tradis | HY |
| PA0210 | *mdcC* | Tradis | Tradis | CC |
| PA0580 | *gcp* | Both | Tradis | TL |
| PA0768 | *lepB* | Both | Tradis | SE/ TL |
| PA0802 | PA0802 | Tradis | Tradis | ME |
| PA0850 | PA0850 | Tradis | Tradis | HY |
| PA1010 | *dapA* | Tradis | Tradis | AA |
| PA1042 | PA1042 | Tradis | Tradis | ME |
| PA1054 | *shaA* | Transit | Transit | ME/ TS |
| PA1059 | *shaF* | Tradis | Tradis | ME/ TS |
| PA1133 | PA1133 | Tradis | Tradis | HY |
| PA1176 | *napF* | Tradis | Tradis | EM |
| PA1255 | *lhpK* | Tradis | Tradis | AA |
| PA1264 | PA1264 | Tradis | Tradis | RE |
| PA1275 | *cobD* | Transit | Tradis | BC |
| PA1314 | PA1314 | Tradis | Tradis | HY |
| PA1549 | PA1549 | Transit | Transit | ME/ TS |
| PA1685 | *masA* | Tradis | Tradis | AA |
| PA1704 | *pcrR* | Tradis | Tradis | RE |
| PA1715 | *pscB* | Tradis | Tradis | SE |
| PA1874 | PA1874 | Transit | Transit | AN |
| PA1908 | PA1908 | Tradis | Tradis | ME/ TS |
| PA1924 | PA1924 | Tradis | Tradis | HY |
| PA1976 | *ercS'* | Transit | Transit | TC |
| PA1988 | *pqqD* | Tradis | Tradis | BC |
| PA2034 | PA2034 | Tradis | Tradis | HY |
| PA2094 | PA2094 | Tradis | Tradis | ME/ RE |
| PA2112 | PA2112 | Tradis | Tradis | HY |
| PA2175 | PA2175 | Tradis | Tradis | HY |
| PA2261 | PA2261 | Tradis | Tradis | CC |
| PA2299 | PA2299 | Tradis | Tradis | RE |
| PA2330 | PA2330 | Tradis | Tradis | HY |
| PA2331 | PA2331 | Tradis | Tradis | ME |
| PA2346 | PA2346 | Transit | Tradis | EN |
| PA2368 | *hsiF3* | Tradis | Tradis | HY |
| PA2375 | PA2375 | Tradis | Tradis | ME |
| PA2404 | *fpvH* | Tradis | Tradis | ME |
| PA2405 | *fpvJ* | Tradis | Tradis | HY |
| PA2433 | PA2433 | Tradis | Tradis | HY |
| PA2436 | PA2436 | Tradis | Tradis | HY |
| PA2542 | PA2542 | Transit | Transit | HY |
| PA2805 | PA2805 | Tradis | Tradis | HY |
| PA2851 | *efp* | Tradis | Tradis | TL |
| PA2876 | *pyrF* | Tradis | Tradis | NU |
| PA2910 | PA2910 | Tradis | Tradis | ME |
| PA2916 | PA2916 | Tradis | Tradis | ME |
| PA3005 | *nagZ* | Tradis | Tradis | AA/ AN |
| PA3133.3 | PA3133.3 | Tradis | Tradis | NC |
| PA3417 | PA3417 | Transit | Tradis | EN |
| PA3505 | PA3505 | Tradis | Tradis | HY |
| PA3527 | *pyrC* | Both | Tradis | NU |
| PA3952 | PA3952 | Tradis | Tradis | HY |
| PA4225 | *pchF* | Transit | Transit | SF/ TS |
| PA4395 | PA4395 | Tradis | Tradis | HY |
| PA4457 | PA4457 | Tradis | Tradis | SF |
| PA4531 | PA4531 | Tradis | Tradis | HY |
| PA4673.1 | PA4673.1 | Tradis | Tradis | NC |
| PA4758 | *carA* | Transit | Transit | AA/ NU |
| PA4760 | *dnaJ* | Transit | Tradis | AP/ DN/ CH |
| PA4854 | *purH* | Both | Both | NU |
| PA4855 | *purD* | Transit | Both | NU |
| PA5004 | *wapH* | Transit | Tradis | EN |
| PA5015 | *aceE* | Transit | Both | AA/EN |
| PA5160.1 | PA5160.1 | Tradis | Tradis | NC |
| PA5320 | *coaC* | Tradis | Tradis | BC/DN |
| PA5366 | *pstB* | Transit | Tradis | ME/TS |
| PA5406 | PA5406 | Tradis | Tradis | HY |
| PA5426 | *purE* | Tradis | Tradis | NU |
| PA5565 | *gidA* | Transit | Transit | CD |

**Supplementary Table 4. Genes essential in murine abscess and human skin model but not *in vitro* in physiologically-relevant media conditions.** Genes also essential in MHB have been removed. **Functional Classes** are: AA - Amino acid metabolism and biosynthesis; AN - Antibiotic resistance and susceptibility; AP - Adaptation and protection; BC - Biosynthesis, cofactors, prosthetic groups and carriers; CC - Carbon compound catabolism; CT - Chemotaxis; CM - Central intermediary metabolism; CD - Cell division; CW - Cell wall, LPS, capsule; CH - Chaperones and heat shock proteins; DN - DNA replication and repair; EM – Energy metabolism; EN - Putative enzyme; FA - Fatty acid and phospholipid metabolism; HY - Hypothetical unclassified; ME - Membrane; MO - Motility; NU - Nucleotide biosynthesis and metabolism; PH - Related to phage; RE - Transcriptional regulator; SE - Protein secretion and export; SF - Secreted factors; TC - Two component regulator; TL - Translation and post translational modification; TR - Transcription, RNA processing and degradation; TS - Transport small molecules.

| **locus Tag** | **Gene** | **Description** | **Source Abscess** | **Source skin** | **Functional Class** |
| --- | --- | --- | --- | --- | --- |
| PA2703 | *tsi2* | TypeVI secretion protein | Tradis | Tradis | AP |
| PA3865.1 | PA3865.1 | pyocin S4 immunity protein | Tradis | Tradis | AP |
| PA2920 | PA2920 | probable chemotaxis transducer | Tradis | Tradis | AP/CT |
| PA4844 | *ctpL* | Chemotactic transducer protein | Transit | Transit | AP/CT |
| PA1486 | *bapF* | beta-peptidyl aminopeptidase | Tradis | Tradis | AA |
| PA2442 | *gcvT2* | glycine cleavage system protein T2 | Tradis | Tradis | AA/ CM |
| PA2964 | *pabC* | 4-amino-4-deoxychorismate lyase | Tradis | Tradis | BC |
| PA5563 | *soj* | chromosome partitioning protein | Tradis | Tradis | CD |
| PA2239 | *pslI* | Psl polysaccharide biosynthesis protein | Tradis | Tradis | CW |
| PA4626 | *hprA* | glycerate dehydrogenase | Tradis | Tradis | CM |
| PA1793 | *ppiB* | peptidyl-prolyl cis-trans isomerase B | Tradis | Tradis | CH/TL |
| PA0965 | *ruvC* | Holliday junction resolvase | Tradis | Tradis | DN |
| PA0967 | *ruvB* | Holliday junction DNA helicase | Tradis | Tradis | DN |
| PA5345 | *recG* | ATP-dependent DNA helicase | Transit | Transit | DN/RE |
| PA2965 | *fabF1* | beta-ketoacyl-acyl carrier protein synthase II | Tradis | Tradis | FA |
| PA0332 | PA0332 | hypothetical protein | Tradis | Tradis | HY |
| PA0553 | PA0553 | hypothetical protein | Tradis | Tradis | HY |
| PA0760 | PA0760 | conserved hypothetical protein | Tradis | Tradis | HY |
| PA1111 | PA1111 | hypothetical protein | Tradis | Tradis | HY |
| PA1263 | PA1263 | hypothetical protein | Tradis | Tradis | HY |
| PA1401 | PA1401 | hypothetical protein | Tradis | Tradis | HY |
| PA1463 | PA1463 | hypothetical protein | Tradis | Tradis | HY |
| PA1492 | PA1492 | hypothetical protein | Tradis | Tradis | HY |
| PA1548 | PA1548 | conserved hypothetical protein | Tradis | Tradis | HY |
| PA1558 | PA1558 | hypothetical protein | Tradis | Tradis | HY |
| PA1766 | PA1766 | hypothetical protein | Tradis | Tradis | HY |
| PA1842 | PA1842 | hypothetical protein | Tradis | Tradis | HY |
| PA2031 | PA2031 | hypothetical protein | Tradis | Tradis | HY |
| PA2384 | PA2384 | hypothetical protein | Tradis | Tradis | HY |
| PA2406 | *fpvK* | ABC transporter for pyoverdine | Tradis | Tradis | HY |
| PA2412 | PA2412 | conserved hypothetical protein | Tradis | Tradis | HY |
| PA2724 | PA2724 | hypothetical protein | Tradis | Tradis | HY |
| PA2733 | PA2733 | conserved hypothetical protein | Tradis | Tradis | HY |
| PA2972 | PA2972 | conserved hypothetical protein | Tradis | Tradis | HY |
| PA3197 | PA3197 | hypothetical protein | Tradis | Tradis | HY |
| PA3198 | PA3198 | conserved hypothetical protein | Tradis | Tradis | HY |
| PA3259.1 | PA3259.1 | Uncharacterized protein | Tradis | Tradis | HY |
| PA3284 | PA3284 | hypothetical protein | Tradis | Tradis | HY |
| PA3632 | PA3632 | conserved hypothetical protein | Tradis | Tradis | HY |
| PA3800 | PA3800 | conserved hypothetical protein | Tradis | Tradis | HY |
| PA4452 | PA4452 | conserved hypothetical protein | Tradis | Tradis | HY |
| PA4534 | PA4534 | hypothetical protein | Tradis | Tradis | HY |
| PA4816 | PA4816 | hypothetical protein | Tradis | Tradis | HY |
| PA4966 | PA4966 | hypothetical protein | Tradis | Tradis | HY |
| PA5198 | PA5198 | LD-carboxypeptidase | Tradis | Tradis | HY |
| PA5401 | PA5401 | hypothetical protein | Tradis | Tradis | HY |
| PA0738 | PA0738 | conserved hypothetical protein | Tradis | Tradis | ME |
| PA1547 | PA1547 | hypothetical protein | Tradis | Tradis | ME |
| PA4962 | PA4962 | conserved hypothetical protein | Tradis | Tradis | ME |
| PA3900 | *fecR* | Regulator of iron dicitrate transport | Tradis | Tradis | ME/RE |
| PA2933 | PA2933 | probable major facilitator superfamily transporter | Tradis | Tradis | ME/TS |
| PA5070 | *tatC* | transport protein | Tradis | Tradis | ME/TS |
| PA3480 | PA3480 | probable deoxycytidine triphosphate deaminase | Tradis | Tradis | NU |
| PA0678 | *hxcU* | Phosphatase secretion protein | Tradis | Tradis | SE |
| PA0687 | *hxcS* | Phosphatase secretion protein | Tradis | Tradis | SE |
| PA0597 | PA0597 | probable nucleotidyl transferase | Tradis | Tradis | EN |
| PA0853 | PA0853 | probable oxidoreductase | Tradis | Tradis | EN |
| PA3330 | PA3330 | probable short chain dehydrogenase | Tradis | Tradis | EN |
| PA4089 | PA4089 | probable short-chain dehydrogenase | Tradis | Tradis | EN |
| PA4202 | *nmoA* | nitronate monooxygenase | Tradis | Tradis | EN |
| PA0624 | PA0624 | hypothetical protein | Tradis | Tradis | PH |
| PA4585 | *rtcA* | RNA 3'-terminal phosphate cyclase | Tradis | Tradis | TR |
| PA4951 | *orn* | oligoribonuclease | Tradis | Tradis | TR |
| PA0424 | *mexR* | multidrug resistance operon repressor | Tradis | Tradis | RE |
| PA0906 | *alpR* | lysis phenotype repressor | Tradis | Tradis | RE |
| PA2273 | *soxR* | Transcriptional activator | Tradis | Tradis | RE |
| PA4896 | PA4896 | probable sigma-70 factor, ECF subfamily | Tradis | Tradis | RE |

**Supplementary Table 5.** **Genes essential *in vitro* in physiologically-relevant conditions that were not essential in abscess or skin.** Genes also essential in MHB have been removed. **Functional Classes** are: AA - Amino acid metabolism and biosynthesis; AN - Antibiotic resistance and susceptibility; AP - Adaptation and protection; BC - Biosynthesis, cofactors, prosthetic groups and carriers; CC - Carbon compound catabolism; CT - Chemotaxis; CM - Central intermediary metabolism; CD - Cell division; CW - Cell wall, LPS, capsule; CH - Chaperones and heat shock proteins; DN - DNA replication and repair; EM – Energy metabolism; EN - Putative enzyme; FA - Fatty acid and phospholipid metabolism; HY - Hypothetical unclassified; ME - Membrane; MO - Motility; NU - Nucleotide biosynthesis and metabolism; PH - Related to phage; RE - Transcriptional regulator; SE - Protein secretion and export; SF - Secreted factors; TC - Two component regulator; TL - Translation and post translational modification; TR - Transcription, RNA processing and degradation; TS - Transport small molecules.

| **Locus Tag** | **Gene** | **Description** | **Essential in** | | **Functional class** |
| --- | --- | --- | --- | --- | --- |
|  |  |  | **Serum** | **RPMI** |  |
| PA0076 | *tagF1* | Type VI secretion protein | NE | Tradis | SE |
| PA0078 | *tssL1* | Type VI secretion protein | NE | Tradis | SE |
| PA0416 | *chpD* | probable transcriptional regulator | NE | Tradis | CT/RE |
| PA0677 | *hxcW* | Phosphatase secretion protein | NE | Tradis | SE |
| PA0878 | PA0878 | hypothetical protein | NE | Tradis | HY |
| PA1054 | *shaA* | Sodium:proton antiporter | Transit | Transit | ME/ TS |
| PA1170 | PA1170 | conserved hypothetical protein | NE | Tradis | ME |
| PA1172 | *napC* | cytochrome c-type protein | NE | Tradis | EM |
| PA1175 | *napD* | NapD protein of periplasmic nitrate reductase | NE | Tradis | EM |
| PA1235 | PA1235 | probable transcriptional regulator | NE | Tradis | RE |
| PA1255 | *lhpK* | D-hydroxyproline epimerase | Tradis | Tradis | AA |
| PA1261 | *lhpR* | Transcriptional regulator of hydroxypriline catabolism | NE | Tradis | AA/RE |
| PA1269 | PA1269 | probable transcriptional regulator | NE | Tradis | RE |
| PA1270 | PA1270 | hypothetical protein | NE | Transit | HY |
| PA1278 | *cobP* | cobinamide kinase | NE | Tradis | BC |
| PA1350 | PA1350 | hypothetical protein | NE | Tradis | HY |
| PA1352 | PA1352 | conserved hypothetical protein | NE | Tradis | ME |
| PA1397 | PA1397 | probable two-component response regulator | NE | Tradis | RE/TR |
| PA1416 | PA1416 | conserved hypothetical protein | NE | Both | HY |
| PA1417 | PA1417 | probable decarboxylase | Transit | NE | EN |
| PA1465 | PA1465 | hypothetical protein | NE | Tradis | HY |
| PA1578 | PA1578 | hypothetical protein | NE | Tradis | HY |
| PA1618 | PA1618 | conserved hypothetical protein | NE | Tradis | HY |
| PA1721 | *pscH* | type III export protein | NE | Tradis | SE |
| PA1869 | PA1869 | probable acyl carrier protein | NE | Tradis | FA |
| PA1870 | PA1870 | hypothetical protein | NE | Tradis | HY |
| PA1874 | PA1874 | hypothetical protein | Transit | Transit | AN |
| PA1891 | PA1891 | hypothetical protein | NE | Tradis | ME |
| PA1960 | PA1960 | hypothetical protein | NE | Tradis | ME |
| PA1972 | PA1972 | conserved hypothetical protein | Transit | NE | ME |
| PA1979 | *eraS* | sensor kinase | NE | Tradis | TC |
| PA2023 | *galU* | UTP--glucose-1-phosphate uridylyltransferase | Transit | NE | CM |
| PA2034 | PA2034 | hypothetical protein | Tradis | Tradis | HY |
| PA2038 | PA2038 | hypothetical protein | NE | Tradis | HY |
| PA2052 | *cynS* | cyanate lyase | NE | Tradis | CM |
| PA2094 | PA2094 | probable transmembrane sensor | Tradis | Tradis | ME/RE |
| PA2115 | PA2115 | probable transcriptional regulator | NE | Tradis | RE |
| PA2120 | PA2120 | hypothetical protein | NE | Tradis | HY |
| PA2143 | PA2143 | hypothetical protein | NE | Tradis | HY |
| PA2190 | PA2190 | conserved hypothetical protein | NE | Tradis | HY |
| PA2249 | *bkdB* | branched-chain alpha-keto acid dehydrogenase (lipoamide component) | NE | Tradis | AA |
| PA2256 | *pvcC* | paerucumarin biosynthesis protein | NE | Transit | AA/SF |
| PA2267 | PA2267 | probable transcriptional regulator | NE | Tradis | RE |
| PA2274 | PA2274 | hypothetical protein | NE | Tradis | HY |
| PA2284 | PA2284 | hypothetical protein | NE | Tradis | HY |
| PA2292 | PA2292 | hypothetical protein | NE | Tradis | HY |
| PA2301 | PA2301 | hypothetical protein | NE | Tradis | HY |
| PA2308 | PA2308 | ATP-binding component of ABC transporter | NE | Tradis | TS |
| PA2351 | PA2351 | probable permease of ABC transporter | NE | Tradis | ME/TS |
| PA2404 | *fpvH* | ABC transporter for pyoverdine | Tradis | Tradis | ME |
| PA2433 | PA2433 | hypothetical protein | Tradis | Tradis | HY |
| PA2905 | *cobH* | precorrin isomerase CobH | Tradis | NE | BC |
| PA2978 | *ptpA* | phosphotyrosine protein phosphatase | NE | Tradis | AP/ CW/ TL |
| PA3073 | PA3073 | hypothetical protein | NE | Tradis | HY |
| PA3090 | PA3090 | hypothetical protein | NE | Tradis | HY |
| PA3178 | PA3178 | hypothetical protein | NE | Tradis | HY |
| PA3209 | PA3209 | conserved hypothetical protein | NE | Tradis | HY |
| PA3214 | PA3214 | hypothetical protein | NE | Tradis | HY |
| PA3406 | *hasD* | transport protein | NE | Transit | SE/ TS |
| PA3411 | PA3411 | hypothetical protein | NE | Tradis | HY |
| PA3413 | PA3413 | conserved hypothetical protein | NE | Tradis | HY |
| PA3415 | PA3415 | probable dihydrolipoamide acetyltransferase | NE | Tradis | EM |
| PA3417 | PA3417 | probable pyruvate dehydrogenase E1 component, alpha subunit | Transit | Tradis | EM |
| PA3458 | PA3458 | probable RE | NE | Tradis | RE |
| PA3727 | PA3727 | hypothetical protein | NE | Tradis | HY |
| PA3763 | *purL* | phosphoribosylformylglycinamidine synthase | Transit | NE | NU |
| PA4020 | *mpl* | UDP-N-acetylmuramate:L-alanyl-gamma-D-glutamyl-meso-diaminopimelate ligase | Transit | NE | AN/CW |
| PA4021 | PA4021 | probable transcriptional regulator | NE | Transit | RE |
| PA4111 | PA4111 | hypothetical protein | NE | Tradis | HY |
| PA4161 | *fepG* | ferric enterobactin transport protein | NE | Tradis | ME/ TS |
| PA4216 | *phzG1* | probable pyridoxamine 5'-phosphate oxidase | NE | Tradis | SF |
| PA4280 | *birA* | BirA bifunctional protein | Transit | NE | BC |
| PA4300 | *tadC* | Type II secretion system protein | NE | Tradis | ME/MO |
| PA4377 | PA4377 | hypothetical protein | NE | Tradis | HY |
| PA4545 | *comL* | competence protein | Transit | NE | CW |
| PA4907 | PA4907 | probable short-chain dehydrogenase | NE | Tradis | EN |
| PA5000 | *wapR* | alpha-1,3-rhamnosyltransferase | Transit | NE | CW |
| PA5005 | PA5005 | probable carbamoyl transferase | Transit | NE | EN |
| PA5221 | PA5221 | probable FAD-dependent monooxygenase | NE | Tradis | EN |
| PA5384 | *hocS* | acylcarnitine hydrolase | NE | Tradis | CC/CM |

Supplementary Table 6. *P. aeruginosa* genes required for survival in the *in vitro* physiologically-relevant media and *in vivo* compared to essentiality in other published studies. ES indicates conditionally required for survival; - indicates not conditionally essential; nd, No data , indicates strains used in the experiment did not have a PAO1 ortholog or was not included in the study.

| **Locus Tag** | **Gene** | **Description** | **Also essential in** | | | |
| --- | --- | --- | --- | --- | --- | --- |
|  |  |  | Wound^a^ | Serum^b^ | SCFM^c^ | Sputum^d^ |
| PA1010 | *dapA* | Lysine biosynthesis via diaminopimelate | - | - | ES | ES |
| PA1685 | *masA* | Methionine salvage pathway enolase-phosphatase E-1 | ES | ES | ES | - |
| PA3166 | *pheA* | Chorismate mutase | - | - | - | - |
| PA3151 | *hisF2* | Histidine metabolism | ES | nd | - | - |
| PA3005 | *nagZ* | Beta-N-acetyl-D-glucosaminidase | - | nd | - | - |
| PA2000 | *dhcB* | Valine, leucine and isoleucine degradation | - | - | - | - |
| PA5015 | *aceE* | Valine, leucine, and isoleucine degradation, pyruvate metabolism | - | - | - | - |
| PA1272 | *cobO* | Cobalamin biosynthetic protein | ES | - | - | - |
| PA1276 | *cobC* | Cobalamin biosynthetic protein | ES | - | - | - |
| PA1275 | *cobD* | Cobalamin biosynthetic protein | ES | - | - | - |
| PA2947 | *cobE* | Cobalamin biosynthetic protein | - | - | - | - |
| PA1277 | *cobQ* | Cobyric acid synthase | - | - | - | - |
| PA1988 | *pqqD* | pyrroloquinoline quinone biosynthesis | - | - | - | - |
| PA0210 | *mdcC* | Malonate decarboxylase delta subunit | ES | - | - | - |
| PA2261 | PA2261 | Pentose phosphate pathway; Probable 2-ketogluconate kinase | - | - | - | - |
| PA0212 | *mdcE* | Malonate decarboxylase gamma subunit | ES | - | - | - |
| PA2516 | *xylZ* | Toluate electron transfer component | ES | - | - | - |
| PA1176 | *napF* | Ferredoxin protein | ES | nd | - | - |
| PA2297 | PA2297 | Probable ferredoxin | ES | - | - | - |
| PA1177 | *napE* | Periplasmic nitrate reductase protein | ES | nd | - | - |
| PA4219 | *ampO* | AmpO | - | nd | - | - |
| PA1057 | *shaD* | sodium/hydrogen antiporter protein | ES | - | ES | - |
| PA1059 | *shaF* | sodium/hydrogen antiporter protein | - | - | ES | ES |
| PA1549 | *copA2* | Copper transport | ES | ES | ES | - |
| PA1908 | PA1908 | probable major facilitator superfamily transporter | ES | nd | - | - |
| PA2092 | PA2092 | probable major facilitator superfamilytransporter | - | nd | - | - |
| PA5366 | *pstB* | ATP-binding component of ABC phosphate transporter | - | - | - | - |
| PA4224 | *pchG* | pyochelin biosynthetic protein PchG | ES | - | - | - |
| PA4225 | *pchF* | pyochelin synthetase | ES | - | - | - |
| PA3527 | *pyrC* | Pyrimidine metabolism gene | - | ES | ES | ES |
| PA3050 | *pyrD* | Pyrimidine metabolism gene | ES | ES | ES | ES |
| PA2876 | *pyrF* | Pyrimidine metabolism gene | ES | ES | ES | ES |
| PA5426 | *purE* | Purine metabolism gene | - | ES | - | ES |
| PA3108 | *purF* | Purine metabolism gene | ES | ES | - | ES |
| PA2991 | *sth* | sol. pyridine nucleotide transhydrogenase | ES | - | ES | - |
| PA4758 | *carA* | carbamoyl-phosphate synthase small chain | - | ES | ES | ES |
| PA3505 | PA3505 | Hypothetical dehydrogenase | ES | ES | ES | ES |
| PA1715 | *pscB* | type III export apparatus protein | - | - | - | - |
| PA5068 | *tatA* | translocation protein | ES | nd | ES | - |
| PA5069 | *tatB* | translocation protein | - | ES | ES | - |
| PA1696 | *pscO* | translocation protein in type III secretion | ES | - | - | - |
| PA0768 | *lepB* | signal peptidase I | ES | ES | ES | ES |
| PA0678 | *hxcU* | Pseudopillin; alk. phosphatase secretion | ES | - | - | - |
| PA0677 | *hxcW* | Pseudopillin; alk. phosphatase secretion | - | - | - | - |
| PA4457 | *kdsD* | Arabinose-5-phosphate isomerase, LPS biosynthesis | ES | ES | ES | ES |

^a^ Murine chronic wound model: Turner et al. 2014; ^b^ Bovine serum: Poulsen et al. 2019; ^c^ Synthetic CF medium: Turner et al. 2015; ^d^ Human Sputum: Lee et al. 2015;

Supplementary Table 7. Fold changes in gene expression under host-mimicking conditions of genes belonging to essential *P. aeruginosa* functional classes in similar conditions. Fold changes were from previous studies: RPMI/serum-grown strain PAO1 by RNA-Seq^a^, Abscess-grown strain LESB58 by qPCR^b^; Chronic and acute wound isolated PAO1 by RNA-Seq^c^.

| **Gene** | **Description** | **FC cf. rich media** | | **FC cf. minimal medium** | |
| --- | --- | --- | --- | --- | --- |
|  |  | **RPMI/ Serum vs. MHB** | **Abscess vs. LB** | **Chronic wound vs. MOPS-succinate** | **Acute wound vs. MOPS-succinate** |
| *pchB* | Pyochelin siderophore biosynthesis | 131 | 8.2 | 15.07 | 54.21 |
| *pvdE* | Pyoverdine siderophore ABC transporter | 340.4 | 124.8 | 6.54 | 11.37 |
| *exsA* | Type III secretion transcriptional activator | 16.2 | 8.0 | 2.35 | 6.62 |
| *pscI* | Type III secretion export protein | 5.9 | -2.5 | 1.73 | 3.99 |
| *toxA* | Exotoxin A type II secretion system substrate | 50.3 | 24.9 | 3.94 | 6.94 |
| *lasB* | Elastase type II secretion system substrate | 25.4 | -2.9 | 1.82 | 13 |
| *lipC* | Lipase type II secretion system substrate | 1 | 13.2 | 9.75 | 4.46 |
| *lipA* | Major lipase type II secretion system substrate | 10.5 | 10.4 | 3.84 | 1.14 |
| *fimT* | Type 4 pilus biogenesis protein | 1 | 17.9 | 5.8 | 2.2 |
| *fliI* | Flagellum-specific ATP synthase | 1 | 5.4 | -2.27 | -1.84 |
| *rhlB* | Rhamnolipid rhamnosyltransferase chain B | 17.1 | -2.6 | 35.31 | 65.01 |
| *pslD* | Psl exopolysaccharide biosynthesis | 1 | 5.4 | -10 | -3.43 |
| *phzA2* | Phenazine biosynthesis | 20.3 | -12.8 | 6.52 | 61.83 |
| *wzz* | LPS O-antigen chain length regulator | 1 | 24.4 | -1.45 | 1.54 |
| *chpE* | Probable chemotaxis protein | -1.9 | 8.2 | 2.88 | 1.49 |
| *nirQ* | Denitrification regulatory protein | 1.6 | -1.2 | 1.99 | 2.49 |
| *algR* | Global two-component response regulator | 2.9 | -1.6 | 1.8 | 2.94 |
| *gacA* | Two-component system response regulator | 1.9 | -14.6 | -1.83 | -1.11 |
| *mucC* | Positive regulator alginate biosynthesis | 1 | 6.8 | -2.78 | -1.6 |
| *relA* | Stringent stress mediator ppGpp synthesis | 1 | 1.2 | -3.7 | -2.34 |

^a^Belanger et al., 2020; ^b^ Pletzer et al, 2017, ^c^ Turner et al., 2014.

Supplementary Table 8. Components of RPMI-1640 and human serum in µM. RPMI components are from ThermoFisher.

| **Category** | **Compound** | **RPMI-1640** | **Serum*** |
| --- | --- | --- | --- |
| Inorganic salts | NO_3_^-^ | 880 | 10-40 |
|  | Ca^++^ | 420 | 2100-2700 |
|  | K^+^ | 536.5 | 5000 |
|  | Cl^-^ | 108,034 | 103,000 |
|  | Mg^++^ | 410 | 823 |
|  | SO_4_^-^ | 410 | 163 |
|  | Na^+^ | 109,367 | 137,400 |
|  | PO_4_^-^ | 6,667 | 337 |
|  | NH_4_^+^ (NH_3_) | NA | < 29 |
|  | Fe | NA | 18-30 |
| Amino acids | L-Arginine HCl | 949 | 60-100 |
|  | L-Alanine | NA | 215-400 |
|  | L-Asparagine | 378 | 45-130 |
|  | L-Aspartic acid | 150 | 0-6 |
|  | Betaine | NA | 50-75 |
|  | L-Carnitine | NA | 43-50 |
|  | L-Cystine | 208 | 30-65 |
|  | L-Glutamic acid | 136 | 18-98 |
|  | L-glutamine | 2,053 | 350-600 |
|  | Glycine | 133 | 250-400 |
|  | L-Histidine FB | 96 | 85-130 |
|  | L-Isoleucine | 381 | 40-70 |
|  | L-lactic acid | NA | 888-1,888 |
|  | L-Leucine | 381 | 110-160 |
|  | L-Lysine | 274 | 150-220 |
|  | Malonic acid | NA | 10-15 |
|  | L-Methionine | 101 | 25-35 |
|  | Methanol | NA | 60-80 |
|  | L-Ornithine | NA | 30-80 |
|  | L-Phenylalanine | 91 | 60-95 |
|  | L-Proline | 174 | 110-350 |
|  | Pyruvic acid | NA | 35-130 |
|  | L-Serine | 285 | 100-165 |
|  | L-Threonine | 168 | 92-240 |
|  | L-Tryptophan | 24 | 45-65 |
|  | L-Tyrosine | 159 | 45-103 |
|  | Taurine | NA | 40-130 |
|  | L-Valine | 170 | 150-310 |
| Vitamins | d-Biotin (Vitamin B_7_) | 0.82 | 0.05 |
|  | D-Ca Pantothenate | 1.14 | 0.54 |
|  | Choline Chloride | 21 | 15-100 |
|  | Folic Acid | 2.3 | 0.04 |
|  | Myo-Inositol | 194 | 20-260 |
|  | Nincotinamide (Vitamin B_3_) | 8.19 | 2.45 |
|  | Pyridoxine HCl (Vitamin B_6_) | 4.8 | 0.023 |
|  | Riboflavin | 0.53 | 0.02 |
|  | Thiamine HCl | 2.9 | 0.02 |
|  | Cobalamin (Vitamin B_12_) | 0.004 | 0.00015 |
|  | citrulline | NA | 16-55 |
|  | citric acid | NA | 100-140 |
|  | D-Glucose | 11,100 | 3,900-7,000 |
| Other | Para-Aminobenzoic Acid | 7.3 | 20-35 |
|  | Glutathione | 3.3 | 941 |
|  | HEPES | 25,000 | NA |
|  | NaHCO3 | 32,700 | 37,000 |
|  | urea | NA | 4,395 |
|  | 2-Hydroxybutyric acid (alpha) | NA | 30-48 |
|  | 3-Hydroxybutyric acid (beta) | NA | 700-800 |
|  | acetic acid | NA | 25-60 |
|  | acetone | NA | 20-70 |
|  | creatine | NA | 15-81 |
|  | creatinine | NA | 70-98 |

*Human serum components are expressed as averages or ranges calculated from (Corso et al., 2017; Fukuda et al., 1984; Krebs, 1950).

Supplementary Table 9. Genes involved in amino acid catabolism and alternative mechanisms of nucleotide synthesis in *P. aeruginosa* were upregulated in RPMI/Serum cf. MHB. Fold changes (FC) determined by RNA-Seq obtained from Belanger at al., 2020.

| **Locus Tag** | **Gene** | **Description** | **FC RPMI/Serum cf. MHB** | **FC RPMI cf. MHB** |
| --- | --- | --- | --- | --- |
| PA5173 | *arcC* | carbamate kinase | 2.8 | 1.5 |
| PA1337 | *ansB* | glutaminase-asparaginase | 2.2 | 1 |
| PA4397 | *panE* | ketopantoate reductase | 2.4 | 2.8 |
| PA1566 | *pauA3* | Catabolism of glutamate | 2.6 | 1 |
| PA2040 | *pauA4* | Catabolism of glutamate | 2.7 | 3.4 |
| PA3356 | *pauA5* | Catabolism of glutamate | 2.4 | 2.9 |
| PA5522 | *pauA6* | Catabolism of glutamate | 2.0 | 2.3 |
| PA5508 | *pauA7* | Catabolism of glutamate | 4.7 | 2.9 |
| PA2776 | *pauB3* | Catabolism of glutamate | 2.2 | 2.3 |
| PA5312 | *pauC* | Catabolism of glutamate | 2.8 | 2.7 |
| PA1742 | *pauD2* | Catabolism of glutamate | 2.3 | 2.3 |
| PA5091 | *hutG* | Histidine to glutamate catabolism | 2.1 | 4.4 |
| PA5092 | *hutI* | Histidine to glutamate catabolism | 2.0 | 3.9 |
| PA5093 | PA5093 | Histidine to glutamate catabolism | 2.1 | 5.4 |
| PA5094 | PA5094 | Histidine to glutamate catabolism | 2.0 | 5.4 |
| PA5095 | PA5095 | Histidine to glutamate catabolism | 2.5 | 6.2 |
| PA5096 | PA5096 | Histidine to glutamate catabolism | 4.3 | 13.1 |
| PA5097 | PA5097 | Histidine to glutamate catabolism | 1 | 13.1 |
| PA5098 | *hutH* | Histidine to glutamate catabolism | 14.7 | 14.7 |
| PA5099 | PA5099 | Histidine to glutamate catabolism | 14.8 | 14.8 |
| PA5100 | *hutU* | Histidine to glutamate catabolism | 3.4 | 26.9 |

**References**

Belanger, C. R., Lee, A. H.-Y., Pletzer, D., Dhillon, B. K., Falsafi, R., and Hancock, R. E. W. (2020). Identification of novel targets of azithromycin activity against *Pseudomonas aeruginosa* grown in physiologically relevant media. *Proc. Natl. Acad. Sci.* 117, 33519–33529. doi:10.1073/pnas.2007626117.

Lee, S. A., Gallagher, L. A., Thongdee, M., Staudinger, B. J., Lippman, S., Singh, P. K., et al. (2015). General and condition-specific essential functions of *Pseudomonas aeruginosa*. *Proc. Natl. Acad. Sci.* 112, 5189–5194. doi:10.1073/pnas.1422186112.

Liberati, N. T., Urbach, J. M., Miyata, S., Lee, D. G., Drenkard, E., Wu, G., et al. (2006). An ordered, nonredundant library of *Pseudomonas aeruginosa* strain PA14 transposon insertion mutants. *Proc. Natl. Acad. Sci.* 103, 2833–2838. doi:10.1073/pnas.0511100103.

Pletzer, D., Wolfmeier, H., Bains, M., and Hancock, R. E. W. (2017). Synthetic peptides to target stringent response-controlled virulence in a *Pseudomonas aeruginosa* murine cutaneous infection model. *Front. Microbiol.* 8, 1867. doi:10.3389/fmicb.2017.01867.

Poulsen, B. E., Yang, R., Clatworthy, A. E., White, T., Osmulski, S. J., Li, L., et al. (2019). Defining the core essential genome of *Pseudomonas aeruginosa*. *Proc. Natl. Acad. Sci.* 116, 10072–10080. doi:10.1073/pnas.1900570116.

Turner, K. H., Everett, J., Trivedi, U., Rumbaugh, K. P., and Whiteley, M. (2014). Requirements for *Pseudomonas aeruginosa* acute burn and chronic surgical wound infection. *PLOS Genet.* 10, e1004518. doi:10.1371/journal.pgen.1004518.

Turner, K. H., Wessel, A. K., Palmer, G. C., Murray, J. L., and Whiteley, M. (2015). Essential genome of *Pseudomonas aeruginosa* in cystic fibrosis sputum. *Proc. Natl. Acad. Sci.* 112, 4110–4115. doi:10.1073/pnas.1419677112.

Skurnik, D., Roux, D., Aschard, H., Cattoir, V., Yoder-Himes, D., Lory, S., et al. (2013). A Comprehensive analysis of in vitro and in vivo genetic fitness of *Pseudomonas aeruginosa* Using High-Throughput Sequencing of Transposon Libraries. *PLoS Pathog.* 9, e1003582. doi:10.1371/journal.ppat.1003582.
